# Kinetics vs Thermodynamics: Engineering Photoredox Reactivity from an Upper Excited State of Fe^II^

**DOI:** 10.1021/jacs.6c02681

**Published:** 2026-06-04

**Authors:** Atanu Ghosh, Björn Pfund, Jonathan T. Yarranton, Yi-Jyun Lien, James K. McCusker

**Affiliations:** Department of Chemistry, 3078 Michigan State University, 578 South Shaw Lane, East Lansing, Michigan 48824, United States

## Abstract

Ultrafast deactivation of metal-to-ligand charge-transfer (MLCT) states into low-lying metal-centered states has long limited the utility of first-row transition-metal complexes in a broad range of applications, including photoredox catalysis. Here we bypass such limitations using a Fe^II^-pyridinium carbene complex to enable electron transfer reactivity directly from a higher-lying MLCT state. Time-resolved spectroscopic data revealed that the higher-energy MLCT manifold relaxes to lower-lying metal-centered states with a time constant of ca. 3 ps. The nature of the metal-centered (MC) excited state as a ^3^MC (i.e., *S* = 1) was inferred from variable-temperature transient absorption studies. These experiments also revealed that the ground-state recovery process occurs at or very near the “Marcus barrierless regime”, thereby allowing for an estimate of its excited-state redox potential. The detailed picture of the energetics of this compound that emerged enabled fine-tuning of competing thermodynamic and kinetic pathways to effect electron transfer from the higher-energy MLCT excited state prior to relaxation to the ligand-field manifold. We believe these results open an unexplored landscape for the use of earth-abundant first-row transition-metal-based chromophores for applications in light-to-chemical energy conversion.

## Graphical abstract

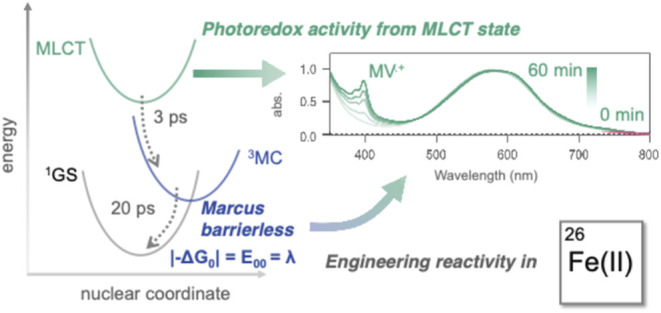

## Introduction

Photoredox catalysis has emerged as one of the most versatile tools in modern synthesis, enabling a broad range of bond-forming reactions by harnessing visible-light energy.^1,2^ Although the field has been dominated by the success of Ru^II^ and Ir^III^ polypyridyl complexes, earth-abundant Fe^II^ complexes offer an appealing substitute for such well-established yet elementally scarce systems in light energy conversion applications, including solar cells and photoredox catalysis.^3,4^ Their strong visible-light absorption and rich redox chemistry — key properties in photochemistry are, however, often overshadowed by their intrinsically weaker ligand fields. This results in the stabilization of metal-centered (MC) states,^5^ which in turn deactivates the metal-to-ligand charge-transfer (MLCT) state on a subpicosecond time scale (Figure 1A),^6–8^ orders of magnitude too short for diffusion-based bimolecular photochemistry. This deactivation pathway usually funnels into a ^5^MC state, which is significantly longer-lived,^6,9,10^ but stores little energy and requires large reorganization energy for electron transfer, leaving it effectively redox-inactive.^11^

**Figure 1:**
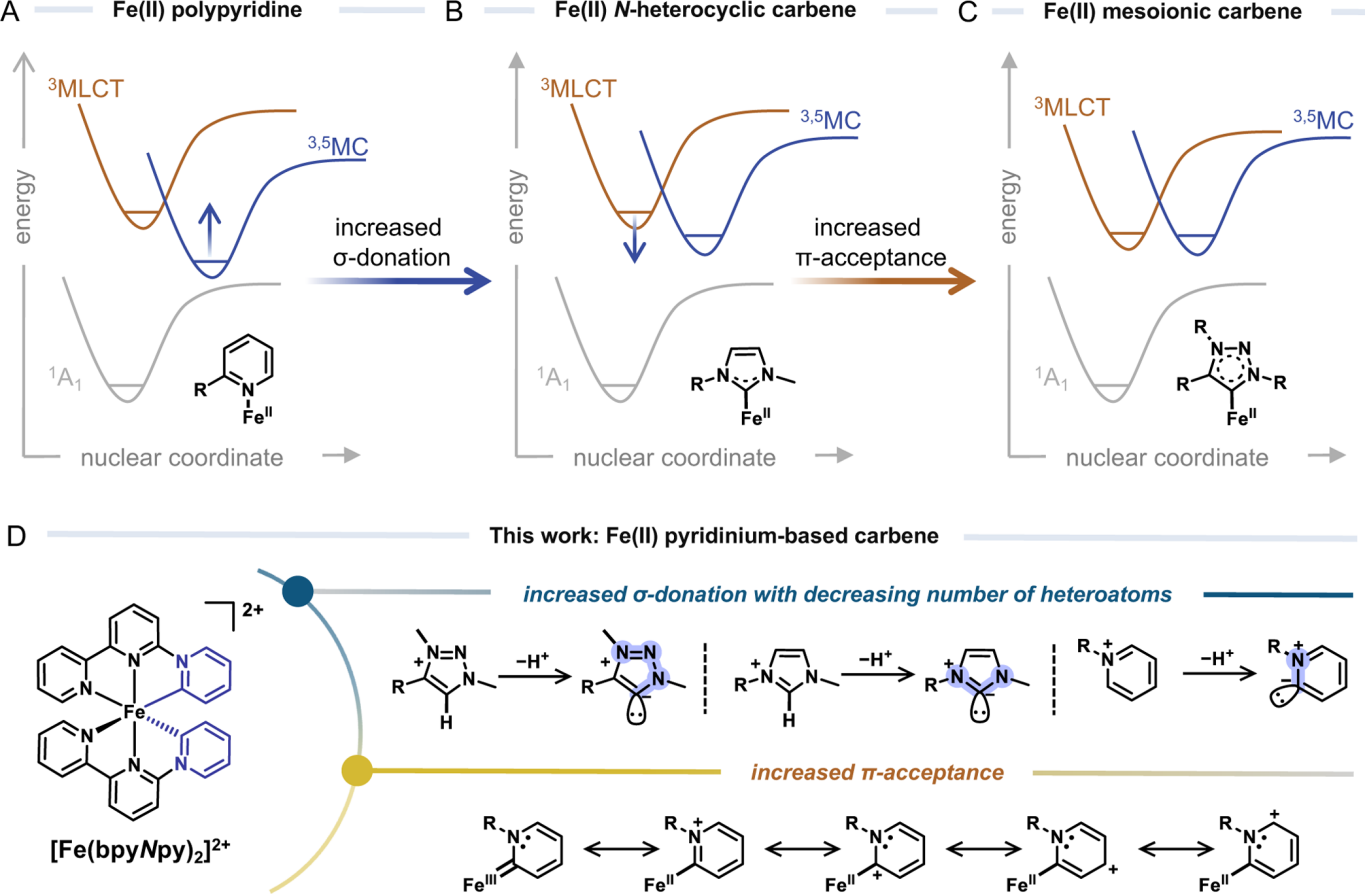
Design Principle of a Modular Fe^II^-pyridinium Carbene. (A-C) Schematic representation of the potential-well energy diagrams for Fe^II^-polypyridine (A), Fe^II^ *N*-heterocyclic carbene (B), and Fe^II^ mesoionic carbene (C) complexes, highlighting the relative energies of the relevant metal-to-ligand charge-transfer (MLCT) and metal-centered (MC) excited states. (D) Molecular structure of the modular Fe^II^-pyridinium carbene complex used for this study, together with the design strategy aimed to destabilize the MC states and stabilize the MLCT manifold through strong σ-donation and increased π-acceptance properties.

To suppress the ultrafast MLCT deactivation, two main design strategies have been pursued. One approach aims to identify and restrict the nuclear motions that are actively coupled to the MLCT deactivation process.^12^ While conceptually intriguing,^13^ rationally designing such ligands that enforce restrictions along specific normal mode vibrations has proven extremely challenging.^14^ The more commonly invoked strategy aims to destabilize MC states by introducing stronger ligand fields.^15^ A landmark advance came in 2013, when the team of Wärnmark and Sundström introduced strongly σ-donating *N*-heterocyclic carbenes (NHCs) to Fe^II^, extending the MLCT lifetime to the picosecond regime, significantly longer than in typical Fe^II^ polypyridyls (Figure 1B).^16^ Several strategies, including cyclometalation and HOMO inversion, have emerged since then to lengthen MLCT lifetimes of Fe^II^ complexes.^17–20^ More recently, mesoionic carbenes, combining strong σ-donation with enhanced π-acceptance, have pushed MLCT state lifetimes to ∼500 ps (Figure 1C).^21^ This ligand design not only destabilizes MC states but also lowers the MLCT state, further hindering the undesired MLCT-to-MC deactivation. Despite these and other advances,^22–28^ the longest-lived MLCT states in Fe^II^ are still below the nanosecond regime: with only a few exceptions,^29,30^ this time scale represents an important benchmark for conventional bimolecular photoredox chemistry in solution.

These limitations have prompted investigations of other first-row transition metal-based complexes such as V^II^,^31^ Cr^0^,^32^ Mn^I^,^33^ Fe^III^,^34–37^ Co^III^ ^38–45^ and Ni^II^,^46–48^ where analogous ligand design has been applied to achieve photochemical reactivity. The impressive progress in the field of first-row transition-metal photochemistry over the past decade notwithstanding,^34,42,49–51^ Fe^II^ carbene complexes remain uniquely appealing for photophysical studies because of their accessible spin-state landscape and natural elemental abundance, but their short excited-state lifetimes have reinforced the prevailing view that Fe^II^ photosensitizers are inherently unsuitable for productive photochemistry. Recently, however, it has been demonstrated that static preassociation of photocatalysts with substrates can drive electron transfer on the picosecond time scale. Notable examples include halide oxidation using ion-pairing,^52,53^ electron transfer through favorable Coulombic interaction,^54–56^ proximity-enabled solvent oxidation or reduction,^57,58^ and electron transfer from organic radicals.^59–62^ Building on these concepts, we posited that preassociation could enable productive photochemistry from the higher-lying MLCT state of Fe^II^, thereby outcompeting its ultrafast deactivation to metal-centered states and the associated loss of stored energy. While photosubstitution reactions prior to excited-state thermalization have been investigated previously,^63^ we wanted to initiate productive photochemical pathways that offers the potential to drive organic photoredox reactions. To test this idea, we designed and synthesized a modular Fe^II^-carbene complex and investigated its photophysical properties. Our results provide insights into the elusive photophysics of this class of chromophores, including the spin-state nature of the metal-centered states. Semiclassical Marcus theory analyses revealed a *Marcus barrierless* ground state recovery process, which was leveraged to estimate the excited state redox potential of the nonemissive metal-centered state. Using these energetic insights as a foundation, we engineered picosecond photoredox reactivity from the higher-energy MLCT state prior to energy loss via conversion to lower-lying metal-centered states through preorganization of the substrate and chromophore. We believe this work demonstrates that photochemical transformations can be initiated from a higher-energy excited state before significant energy loss occurs, opening potential new avenues for first-row transition metal-based photochemistry.

## Results and Discussion

### Design Principle, Synthesis, and Characterization of a Modular Fe^II^-carbene: [Fe­(bpy*N*py)]_2_]^2+^

We designed a modular homoleptic Fe^II^-carbene complex, [Fe­(bpy*N*py)_2_]^2+^ (where bpy*N*py is 6-(*N*-pyridinium)-2,2′-bipyridine), where the ligand framework combines a pyridinium carbene with a tunable bipyridine-based scaffold. The bipyridine unit ensures strong visible-light absorption,^32^ whereas the pyridinium carbene enhances donor strength relative to imidazolium carbenes by avoiding resonance stabilization and concurrently introduces π-accepting character,^64–68^ a combination that we believed offered promise for prolonging the higher-lying MLCT state into the picosecond regime.

The ligand precursor, [bpy*N*pyH]­(PF_6_) was synthesized by adapting a previously published procedure (see Supporting Information).^64^ The complexation of 1 equiv of (bpy*N*pyH)­(PF_6_) and 0.5 equiv of FeBr_2_ in the presence of 40 equiv of triethylamine in ethanol at 100 °C for 18 h, followed by metathesis using aqueous potassium hexafluorophosphate and crystallization, resulted in the formation of [Fe­(bpy*N*py)_2_]­(PF_6_)_2_ in 78% yield (Figure 2A). The diamagnetic [Fe­(bpy*N*py)_2_] compound was characterized by NMR spectroscopy, mass spectrometry, and elemental analysis (Supporting Information). Upon formation of [Fe­(bpy*N*py)_2_]^2+^, a new resonance appears at 227.76 ppm in the ^13^C NMR spectrum in CD_3_CN, which can be attributed to the ligating carbon atom of the pyridinium unit. This assignment is consistent with the chemical shift observed for analogous Ru^II^ complexes.^65,66^

**Figure 2:**
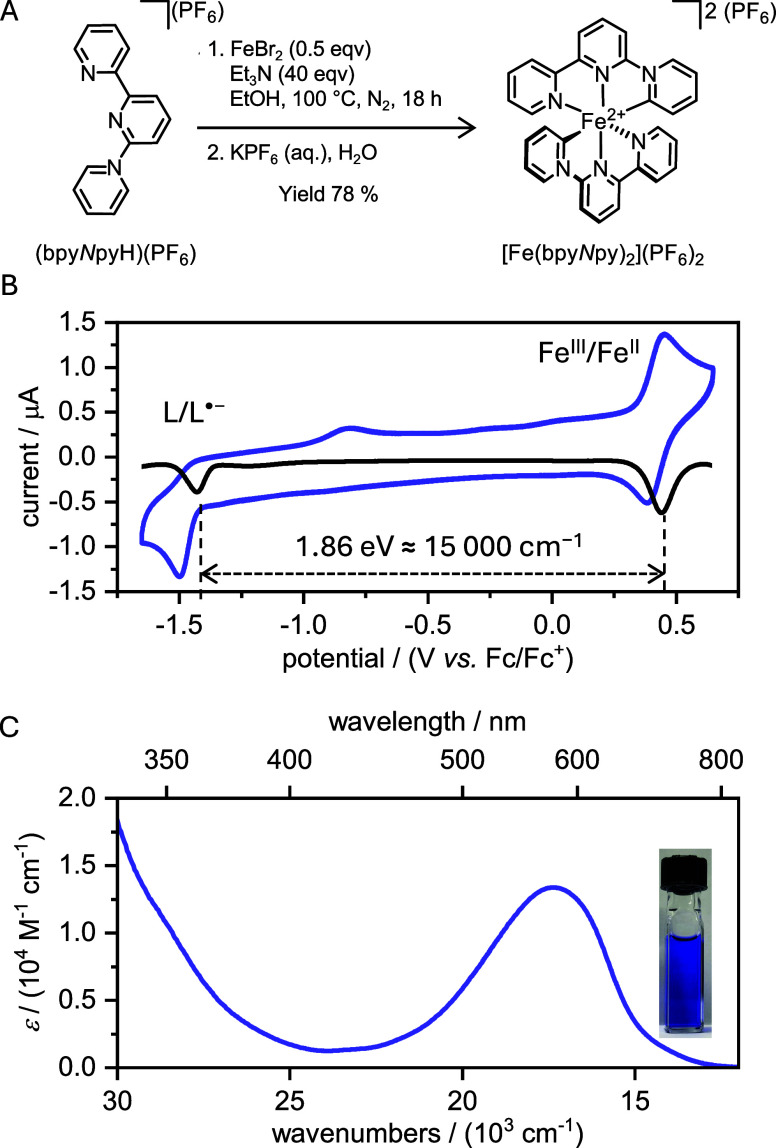
Synthesis and Characterization of [Fe­(bpy*N*py)_*2*_]^2+^. (A) Synthetic scheme for the preparation of the modular pyridinium-based Fe^II^ carbene complex. (B) Cyclic voltammetry (purple line) and differential pulse voltammetry (black line) of 1 mM [Fe­(bpy*N*py)_2_]^2+^ in acetonitrile at 20 °C with 0.1 M (^*n*^Bu_4_N)­(PF_6_) as supporting electrolyte, recorded at a scan rate of 50 mV s^–1^ and externally referenced to the Fc/Fc^*+*^ couple. (C) Electronic absorption spectrum of [Fe­(bpy*N*py)_2_]^2+^ in acetonitrile at 20 °C; Inset: solution of [Fe­(bpy*N*py)_2_]^2+^ in acetonitrile displaying its blue color, a characteristic that is highly unusual for an Fe^II^ complex.

Cyclic and differential pulse voltammetry of [Fe­(bpy*N*py)_2_]^2+^ reveals a reversible oxidation at +0.44 V vs Fc/Fc^+^ (Figure 2A) assigned to a metal-centered oxidation event (i.e., the Fe^II^/Fe^III^ couple). This value matches that of the analogous imidazolium carbene complex [Fe­(bpy^Me^Im)_2_]^2+^ (bpy^Me^Im = 1-(2,2′-bipyridyl)-3-methylimidazol-2-ylidene),^24^ indicating similar character of the Fe^II^ *t*_2g_ orbitals in both complexes. An additional irreversible wave at −1.44 V vs Fc/Fc^+^ is assigned to ligand reduction, shifted 300 mV positively relative to the imidazolium analog. This shift suggests enhanced π-accepting character of the (bpy*N*py)­(PF_6_) ligand when coordinated to Fe^II^, consistent with the positive formal charge on the nitrogen directly connected to the bipyridine ring. As a result, the lowest energy MLCT state of [Fe­(bpy*N*py)_2_]^2+^ is estimated at 1.86 eV, which is approximately 0.3 eV more stabilized relative to the imidazolium analogue.

The electronic absorption spectrum of [Fe­(bpy*N*py)_2_]^2+^ confirms this expectation, where a broad absorption feature centered around 580 nm with a molar absorptivity of 12 000 M^–1^cm^–1^ can be assigned as a spin-allowed ^1^A_1_→^1^MLCT transition (Figure 2C). Compared to the well-known [Fe­(tpy)_2_]^2+^ (where tpy is 2,2′:6′,2″-terpyridine)^67^ and the analogous imidazolium complex ([Fe­(bpy^Me^Im)_2_]^2+^),^24^ the absorption maximum of [Fe­(bpy*N*py)_2_]^2+^ at 580 nm is significantly red-shifted, giving rise to a distinctive blue color to the solution (Figure 2C, inset). This shift originates from the stronger σ-donation and enhanced π-accepting properties of the pyridinium carbene ligand, which makes metal oxidation and ligand reduction energetically more favorable while retaining a large absorption cross-section. The combination of high oscillator strength with a lower-energy MLCT transition renders [Fe­(bpy*N*py)_2_]^2+^ particularly intriguing for photophysical investigations and highlights its potential utility in light-energy conversion applications.

### Excited-State Dynamics

To investigate the excited state dynamics of [Fe­(bpy*N*py)_2_]^2+^, ultrafast time-resolved absorption (TA) measurements were performed on an acetonitrile solution of the compound at 20 °C following ^1^A_1_→^1^MLCT excitation at 555 nm (see Supporting Information). At time delays of less than 2 ps (Figure 3A, red traces), the differential absorption spectra display a strong ground state bleach (GSB) between 470 and 630 nm and two excited-state absorption (ESA) features at 410 nm and red of 630 nm. The overall spectral profile resembles that of the ^3^MLCT excited states of Ru^II^ polypyridine complexes, which include essentially the same two ESA bands at 410 nm and >630 nm together with the MLCT ground state bleach.^68^ To support this assignment, we constructed a 1:1 superposition of spectroelectrochemical difference spectra^69^ obtained for [Fe­(bpy*N*py)_2_]^2+^ in acetonitrile at 0.55 V vs Fc/Fc^+^ (Fe^2+^ → Fe^3+^ oxidation, Figure S5) and −1.60 V vs Fc/Fc^+^ (ligand reduction, Figure S5). The composite spectrum closely reproduces the early time TA spectra and corresponds to [Fe^III^(bpy*N*py^•–^)_2_]^2+^ (Figure 3A). Based on previous reports, the ^1^MLCT→^3^MLCT intersystem crossing process likely occurs within <100 fs,^70–72^ hence we assign this early time species to a ^3^MLCT state.

**Figure 3:**
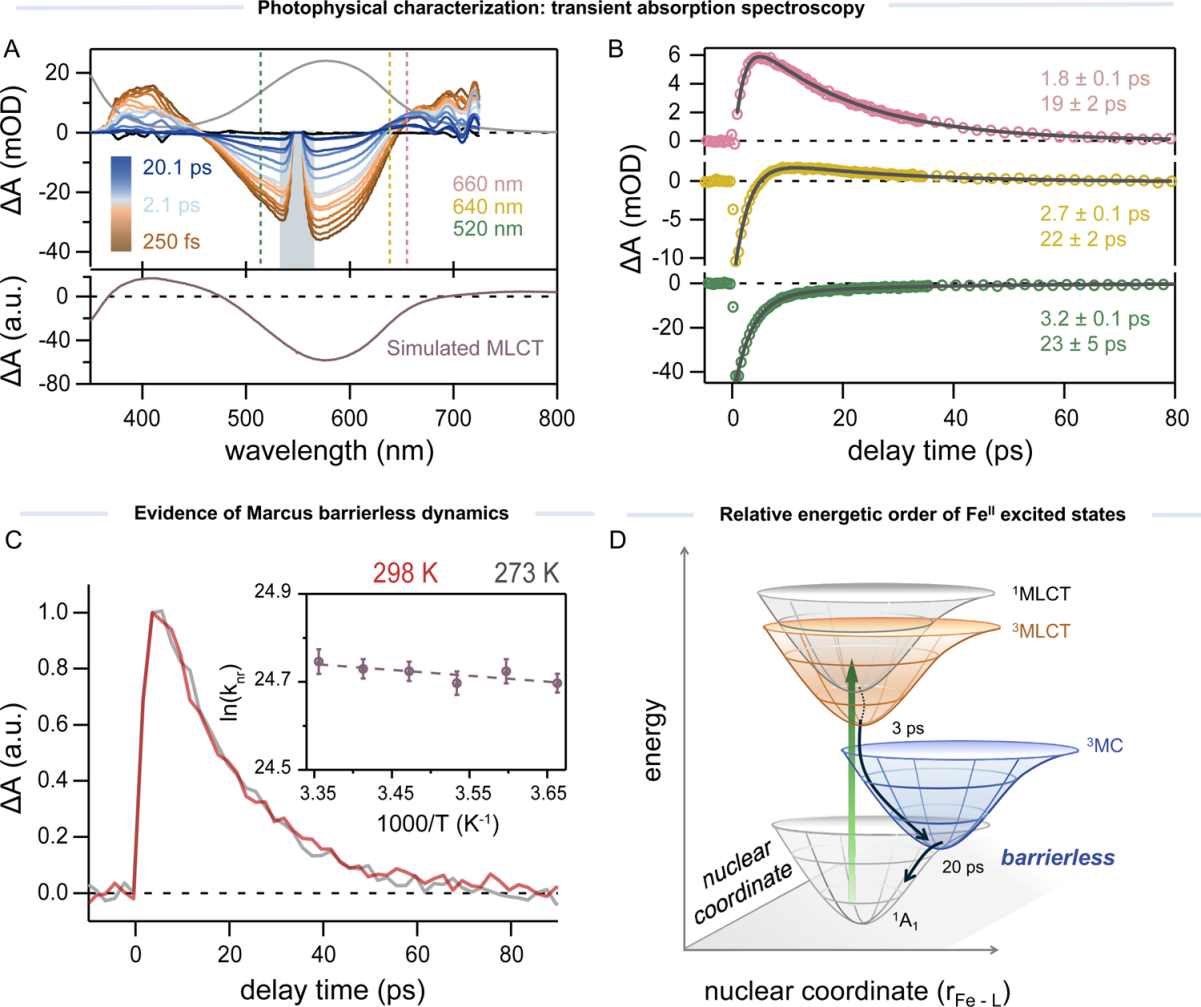
Excited-state Properties of [Fe­(bpy*N*py)_*2*_]^*2+*^. (A) Top: Time-resolved transient absorption (TA) spectra of [Fe­(bpy*N*py)_2_]^2+^ in acetonitrile at 20 °C following photoexcitation at 555 nm (shaded area), recorded at selected delay times. Bottom: Simulated MLCT spectrum obtained from spectroelectrochemical measurements of the oxidized metal center and reduced ligand. (B) Single-wavelength TA kinetics collected at probe wavelengths of 520 nm (bottom), 640 nm (middle), and 660 nm (top) following photoexcitation at 555 nm in acetonitrile at 20 °C. Solid lines represent fits to biexponential kinetic models, yielding the time constants indicated. (C) Ground-state recovery kinetics of [Fe­(bpy*N*py)_2_]^2+^ in acetonitrile, acquired at 660 nm following 555 nm photoexcitation at 273 and 298 K. Inset: Analysis of the variable-temperature ground-state recovery data based on an Arrhenius model. The error bars represent the standard deviation of the data from multiple runs: see the Supporting Information for additional details. (D) Schematic representation of the proposed excited-state landscape of [Fe­(bpy*N*py)_2_]^2+^.

At longer time delays (i.e., time delays greater than 5 ps, Figure 3A, blue traces), the dominant ESA features at 410 nm and >700 nm decay whereas the GSB signal persists. In addition, a new ESA band begins to grow in the range of 630 to 700 nm; these latter two features then persist for more than 20 ps. The complexity of these dynamics, and in particular the evolution of one spectral profile to another, precludes a simple model wherein the compound simply decays from the ^3^MLCT to the ^1^A_1_ ground state. Instead, the observed kinetics require at minimum a model that includes a lower energy MC state (i.e., an electronic excited state devoid of charge-transfer features).

To further support this three-state model, single-wavelength TA kinetics were recorded separately, between 410 and 720 nm (Figure S8); for simplicity, only the kinetic traces at 520, 640, and 660 nm are shown in Figure 3B. At 520 nm, the TA kinetics show GSB recovery that could not be modeled by a single-exponential decay; a biexponential fit, consistent with a three-state (including ground state) model, yielded time constants of 1.8 ± 0.1 and 19 ± 2 ps. Data acquired at 660 nm, which exhibits rising and decaying features, could be fit with the same biexponential. Most significant with respect to the proposed three-state model, however, comes from the 640 nm trace, where a negative GSB rises and evolves into a positive ESA signal within 5 ps after photoexcitation which then persists. Because TA data are plotted as a change in absorbance, a change in sign can only arise from a change in the sign of the molar absorptivity difference between the ground and excited state. Since the ground-state signal is invariant, such a change is unambiguous evidence of evolution from one excited state to another.

Global analysis of the full spectra TA data using a two-component sequential model yielded two-time constants of 2.8 ± 0.4 and 19.6 ± 4 ps, in excellent agreement with our single wavelength kinetic measurements. The evolution-associated spectra (EAS) of the first component (Figure S7) closely resemble the simulated MLCT spectrum (Figure 3A) and is therefore assigned to the ^3^MLCT excited state. The EAS of the second component reveals a substantially different absorption profile and is attributed to a lower-lying MC state that relaxes to the ground state with a time constant of 19.6 ± 4 ps.

#### Identifying the Spin-State Nature of the Metal-Centered Excited State

While the triplet spin character of the MLCT state is readily assigned based on the well-established ultrafast intersystem crossing typical of Fe^II^ complexes, the spin-state nature of the MC states cannot be easily determined from the data just presented, as transient absorption spectroscopy is not an inherently spin-sensitive technique. Time-resolved X-ray absorption and X-ray emission spectroscopy are often employed to directly probe the spin character of MC excited states,^70,73–76^ but these methods require highly specialized and resource-intensive experiments. We recently employed variable-temperature (VT)-TA spectroscopy on a series of Co^III^ polypyridyl complexes to understand their nonradiative ground-state-recovery (GSR) dynamics, which provided insights into the spin character of MC excited states when analyzed through the lens of transition state theory and semiclassical Marcus theory.^41^ We therefore decided to perform VT-TA experiments on [Fe­(bpy*N*py)_2_]^2+^ to better understand its GSR process. One of the important considerations for employing VT time-resolved experiments is ensuring that the dynamics proceed from a thermalized excited state, otherwise thermodynamic parameters obtained from these measurements may lead to erroneous interpretation. We performed additional TA kinetics measurements following 510 nm photoexcitation, which also revealed biexponential decay kinetics with time-constants (Figure S9) similar to those obtained following 555 nm photoexcitation – strongly indicating that the GSR dynamics do proceed from a thermalized metal-centered excited state.

The TA kinetics at 660 nm, where the ESA contribution of the MC state can be isolated, were recorded as a function of temperature from 273 to 298 K in 5 K intervals following photoexcitation at 555 nm. All TA kinetic traces that were acquired can be found in Figure S10; for the sake of clarity, we present only two traces at two extreme temperatures in Figure 3C. The data revealed a minimal change in the GSR lifetime across the entire temperature range, far less that what we have observed in our studies of other first-row metal complexes.^41,67,77^ For comparison, structurally analogous [Fe­(tpy)_2_]^2+^ (where tpy is 2,6:2′,6′:2″-terpyridine) exhibits an easily identifiable temperature dependence, with the GSR lifetime increasing over a comparable temperature range from 4.9 ± 0.2 ns at 295 K to 6.1 ± 0.1 ns at 275 K.^67^ The absence of temperature dependence in the case of [Fe­(bpy*N*py)_2_]^2+^ thus presents a rare example in first-row metal-centered photophysics of a nearly barrierless ground state recovery process. This conclusion is underscored from an Arrhenius analysis of the data (Figure 3C, inset), where the activation energy of 90 ± 40 cm^–1^ is significantly less than the ca. 200 cm^–1^ of thermal energy available at room temperature.

To gain insight into the spin character of the excited state, we fit the data using transition state theory (Figure S11), similar to the approach we adopted previously for both Co^III^ and Fe^II^ complexes.^41,77^ Surprisingly, the Eyring plot exhibits a positive slope, which implies a negative activation enthalpy. This behavior becomes clear upon considering the underlying physical model. As established earlier, ground state recovery dynamics in this system operate in a “barrierless” region, for which the rate constant (*k*_nr_) is expected to show only negligible temperature dependence within transition-state theory. Since Eyring model provides a linear relationship for ln­(*k*_nr_/*T*) vs 1/*T* plot, a decrease in temperature naturally increases the magnitude of the ln­(*k*_nr_/*T*), resulting in a positive slope. The observation of such a slope thus provided additional support for assigning the GSR to a barrierless process. The Eyring plot yielded an activation enthalpy (Δ*H* ^‡^) of –105 ± 35 cm^–1^ and an activation entropy (Δ*S* ^‡^) of −3.65 ± 0.22 cm^–1^ K^–1^. Consistent with the Arrhenius model, Δ*H* ^‡^ was found to be smaller than thermal energy within the measurement temperature range. More relevant to our discussion here, however, is the sign and magnitude of ΔS^‡^, which we have shown can be leveraged to understand the nature of the excited state in question. The negative sign is consistent with a reduction in the molecular volume of the compound upon relaxation from the lowest energy interconfigurational MC state to the ground state. Two possible spin-states for the lowest energy MC state are triplet (^3^T_1_) and quintet (^5^T_2_) which derive from one-electron configurations of (*t*_2g_)^5^(*e*_g_*)^1^ and (*t*_2g_)^4^(*e*_g_*)^2^, respectively; the ^1^A_1_ ground state is represented by (*t*_2g_)^6^(*e*_g_*)^0^. Population of antibonding orbitals in both excited states significantly increases metal–ligand bond length, which correspondingly gives rise to volume contraction upon ground-state recovery. For typical Fe^II^ polypyridyl complexes, where the GSR process proceeds from a ^5^T_2_ ligand-field state, activation entropies have been reported in the range of −5 to −6 cm^–1^ K^–1^.^78,79^ In contrast, for a series of low-spin d^6^-Co^III^ polypyridyl complexes where a ^3^MC state comprises the lowest excited state, a smaller activation entropy of −3 to −4 cm^–1^ K^–1^ has been observed,^41^ i.e., roughly half the magnitude found for the Fe^II^ case; this difference can be qualitatively linked to the difference in e_g_* population between the ^3^T_1_ and ^5^T_2_ states. The observed activation entropy of −3.65 ± 0.20 cm^–1^ K^–1^ for [Fe­(bpy*N*py)_2_]^2+^ is therefore most consistent with a lowest-energy excited state that is triplet in character (i.e., deriving from the ^3^T_1_ excited state) and is indeed similar to the value of −3.20 ± 0.5 cm^–1^ K^–1^ reported for an Fe^II^ phenanthridine-carbene complex that was likewise assigned a ^3^MC state.^77^

#### Estimation of the Zero-Point Energy of the Metal-Centered Excited State

Analyzing the VT data through the lens of semiclassical Marcus theory provides access to the key parameters governing decay, namely the electronic coupling (*H*_ab_) and reorganization energy (λ). In this model, the rate constant is expressed as shown in eq 1,

1 $${k_{\mathrm{nr}}=\frac{2\pi }{\hbar }{\left|H_{ab}\right|}^{2}\frac{1}{\sqrt{4\pi \lambda k_{B}T}}}^{\;}\exp\left\{-\frac{{\left({\Delta G}_{0}+\lambda \right)}^{2}}{4\lambda k_{B}T}\right\}$$

where *k*_nr_ is the decay rate constant obtained from the VT-TA measurements, *k*_B_ is Boltzmann’s constant, *T* is the absolute temperature, ℏ is the reduced Planck constant, and Δ*G*_0_ is the driving force, which in the present circumstance corresponds to the zero-point energy difference between the lowest energy excited state and the ground state. Applying this analysis to [Fe­(bpy*N*py)_2_]^2+^ would, in principle, allow quantification of both λ and H_ab_, thereby connecting the experimental kinetics directly to the underlying electronic structure. In practice, the nonemissive nature of Fe^II^ complexes typically prevents an independent determination of Δ*G*_0_; however, in the present case, the largely temperature-independent nature of the decay rate (Figure 3C) indicates that the GSR process for [Fe­(bpy*N*py)_2_]^2+^ is essentially barrierless. In the context of Marcus theory, this barrierless condition is defined by λ = |Δ*G*_0_|: this cancels the exponential term in eq 1 and leads to a rate constant that is governed entirely by the pre-exponential factor.

Leveraging this insight, we considered a range of reasonable λ values in order to estimate a corresponding range for the magnitude of H_ab_. Previously, the reorganization energy associated with the ^5^MC → ^1^A_1_ ground-state recovery process for the bis-tridentate Fe^II^ complex [Fe­(tpy)_2_]^2+^ had been estimated at ∼14,000 cm^–1^.^13^ In [Fe­(bpy*N*py)_2_]^2+^, however, GSR proceeds from a ^3^MC state. Since the ^5^MC state places two electrons in antibonding *e*_g_* orbitals while the ^3^MC state involves only one, the structural distortion, and thus the reorganization energy, can be estimated to be roughly half as large.^41^ Using a range of 5000–9000 cm^–1^ for λ, the resulting *H*_ab_ values of 20–25 cm^–1^ are much larger than those reported for ^5^MC → ^1^A_1_ GSR in [Fe­(tpy)_2_]^2+^ based systems (3–7 cm^–1^),^67^ where the transition is limited by second-order coupling (Δ*S* = 2) between the quintet excited state and singlet ground state. Instead, the values we estimate for *H*_ab_ in [Fe­(bpy*N*py)_2_]^2+^ closely match those determined for the ^3^MC → ^1^A_1_ process in low-spin d^6^ Co^III^ complexes (16–26 cm^–1^).^41^ Taken together, these comparisons support an assignment of the lowest-energy excited state in [Fe­(bpy*N*py)_2_]^2+^ as a ^3^MC (^3^T_1_) state. A simplified schematic representation of the potential energy surface diagram summarizing the photocycle of the complex is shown in Figure 3D.

### Engineering Photoredox Reactivity from a Higher Excited State

As established from our photophysical studies, the GSR dynamics of [Fe­(bpy*N*py)_2_]^2+^ from the ^3^MC state occur at or near the Marcus barrierless regime, where the driving force equals the reorganization energy (|Δ*G*_0_| = λ). This relationship allows estimation of the zero-point energy difference between the ^3^MC state and the ground state. Based on our estimated range for λ of 5000–9000 cm^–1^, this value is less than 1.0 eV and thus represents a significant energy loss compared to the photoexcited ^1^MLCT state (∼1.9 eV, Figure 2B). Combining the ^3^MC zero-point energy with the ground-state redox potential yields a maximum excited-state potential of –0.56 V vs Fc/Fc^+^, which is far too low for demanding photoredox chemistry. By contrast, analogous estimation of excited state redox potential for the higher energy ^3^MLCT state yielded −1.1 V vs Fc/Fc^+^ (assuming an ∼0.4 eV energy loss from the ^1^MLCT state, similar to the valence-isoelectronic [Ru­(bpy)_3_]^2+^).^80,81^ This potential is sufficiently reducing to initiate productive photoredox transformations.

While conventional photochemical reactivity occurs from the lowest excited state of a given spin multiplicity, previous work has shown that photochemistry can bypass this limitation through anti-Kasha reactivity, where higher-lying excited states of a given spin-multiplicity undergo electron transfer before internal conversion occurs.^82–91^ In such cases, the chromophore retains higher redox driving force than would otherwise be possible. Conceptually, this strategy is extremely powerful, yet most reported examples remain confined to bond homolysis, photoionization, or photosubstitution.^92^ Importantly, truly solution-based systems of catalytic relevance have remained essentially unexplored.

To engineer a proof-of-concept reactivity from the higher energy ^3^MLCT state, we selected methyl viologen (MV^2+^) reduction as a model reaction. Its required reduction potential (−1.0 V vs Fc/Fc^+^) is inaccessible from the low-energy ^3^MC state (0.56 V vs Fc/Fc^+^) but thermodynamically feasible from the ^3^MLCT state (1.1 V vs Fc/Fc^+^). Thus, MV^2+^ reduction would provide unequivocal evidence for reactivity from the higher ^3^MLCT excited state. The challenge, however, lies in the kinetics: the 2 ps lifetime of the ^3^MLCT state is far too short for diffusion-based reactivity. To enable reactivity from such a short-lived higher excited state, preassociation of photosensitizer and substrate is necessary to provide kinetic control over electron transfer and allow the reaction to outcompete intramolecular energy dissipation. Inspired by recent work from Turro and co-workers,^93^ we used aqueous micellar solutions to achieve preassociation. Sodium dodecyl sulfate (SDS) micelles provide a hydrophobic core, that solubilizes [Fe­(bpy*N*py)_2_]^2+^ and a negatively charged surface that electrostatically associates MV^2+^. The interaction of [Fe­(bpy*N*py)_2_]^2+^ with the micelle is evident from its solubility in pure water, while the association of MV^2+^ was confirmed through UV–vis and NMR titration experiments. UV–vis absorption spectra of MV^2+^ were recorded in the absence and presence of SDS, showing clear spectral differences consistent with an interaction with the micelles (Figure S12). We envision that this arrangement places the catalyst and substrate in sufficiently proximity to enable electron transfer to outcompete diffusion and realize reactivity from the higher energy ^3^MLCT state despite its single-digit picosecond lifetime. However, this experiment does not provide information about the MV^2+^ concentration required to achieve maximum preassociation within the SDS micelles. Therefore, NMR titration experiments were performed in D_2_O containing 11 mM SDS (similar to the concentration later used in the photocatalytic system). Incremental addition of MV^2+^ led to systematic shifts of its NMR signals (Figure 4E inset and Figure S22), similar to what has been observed in other studies.^94,95^ At low MV^2+^ concentrations (1–12 mM), relatively large changes in signal positions of MV^2+^ were observed, whereas at higher concentrations (12–100 mM) only minor additional shifts occurred. This behavior indicates that the preassociation equilibrium is largely shifted toward the preassociated species at MV^2+^ concentrations of approximately 10 mM under these micellar conditions.

**Figure 4:**
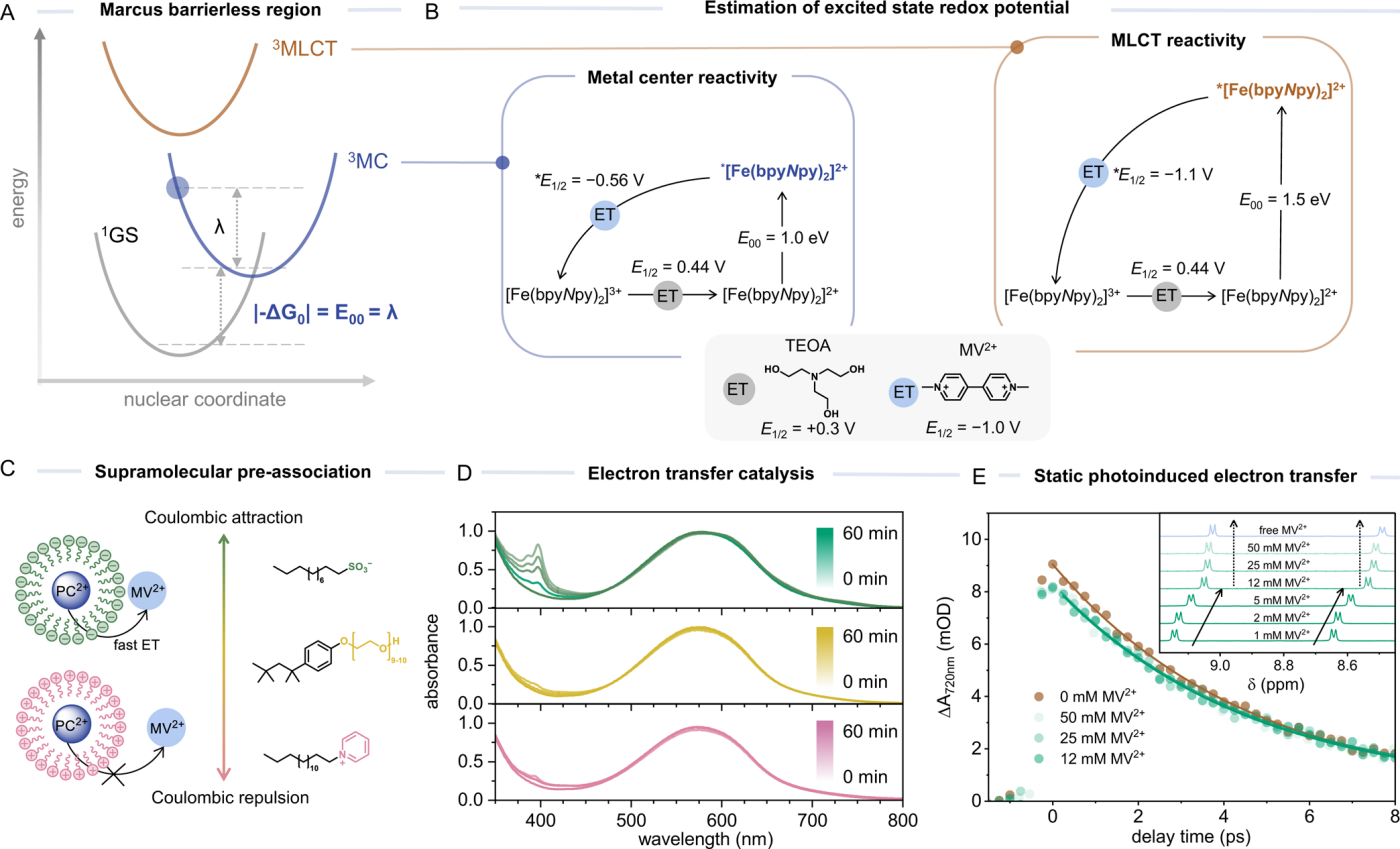
Engineering Photoredox Reactivity from Higher Energy MLCT Excited-states. (A) Simplified schematic representation of the potential energy surface diagram highlighting ground-state-recovery process in the Marcus barrierless region. (B) Estimation of excited state redox potentials of the ^3^MC and ^3^MLCT excited state. (C) Schematic representation of anionic, neutral and cationic micelles used to preorganize photosensitizer and substrate. Control studies were performed with neutral and cationic micelles. (D) Evidence of electron transfer reactivity from higher energy MLCT state. Electronic absorption spectra of [Fe­(bpy*N*py)_2_]^2+^ complex in micellar solution as a function of time in the presence of methyl viologen (MV^2+^) under continuous light irradiation. In the case of an anionic micelle solution, where preassociation of the cationic photosensitizer and cationic substrate are favored, significant electron transfer product features of reduced methyl viologen (MV^•+^) were observed. (E) Ultrafast transient absorption kinetics of [Fe­(bpy*N*py)_2_]^2+^ complex in the absence (brown) and presence (green) of the MV^2+^ quencher in aqueous micellar solution containing 11 mM SDS. The decrease in MLCT signal amplitude upon addition of MV^2+^ indicates static quenching of the charge-transfer excited state, supporting electron transfer from the ^3^MLCT state prior to relaxation to the lower-energy ^3^MC state of the chromophore. Inset: NMR titration experiment of an 11 mM SDS solution in D_2_O upon incremental addition of MV^2+^. The solid arrows indicate pronounced signal shifts, showing that the preassociation equilibrium has not yet been reached, whereas the dotted arrows indicate regions where only minimal additional changes are observed, with the equilibrium being fully shifted toward preassociation of MV^2+^ with SDS micelles.

#### Evidence of Photoredox Reactivity from the MLCT Excited State

With this system in place, a solution containing 80 μM [Fe­(bpy*N*py)_2_]^2+^, 10 mM MV^2+^, 11 mM SDS, and 100 mM TEOA as sacrificial donors was irradiated using a 150 W xenon arc lamp, with the output passed through a monochromator to isolate 560 ± 10 nm light and a long-pass filter to remove wavelengths shorter than 480 nm; progress of the reaction was monitored via steady-state electronic absorption spectroscopy. After 15 min of irradiation, we observed a distinct absorption feature at 395 nm, characteristic of the MV^•+^ product, which continued to increase linearly in intensity with increased irradiation time. The characteristic MV^•+^ absorption bands between 550 and 650 nm are only partially resolved due to the simultaneous decomposition of [Fe­(bpy*N*py)_2_]^2+^. To isolate the MV^•+^ spectrum, the reaction mixture after 1 h of irradiation was exposed to air, reoxidizing MV^•+^ to MV^2+^ via oxygen-mediated back-oxidation. Because MV^2+^ does not absorb in the visible region, subtraction of the spectrum recorded after air exposure from that obtained under inert conditions yields a differential spectrum that closely matches the reference spectrum of MV^•+^ (Figure S16). This unambiguously confirms MV^•+^ formation during photoredox catalysis despite its weakly resolved absorption features at longer wavelengths.

With regard to chromophore decomposition, we speculate a Minisci-type side reaction in the presence of radicals as one of the plausible pathways responsible for the degradation of the photocatalyst.^96,97^ While this side reaction is speculative, this interpretation is consistent with the intrinsic photostability of [Fe­(bpy*N*py)_2_]^2+^ in isolation, for which negligible decomposition products were observed following irradiation under identical conditions (Figures S17 and S24). Importantly, the linearity of product formation (Figure S15) demonstrates that the photochemical reaction itself is not influenced by photoactive decomposition products.^98^ Further control experiments (without photocatalyst, TEOA, or light) remained negative (Figure S13), further indicating ^3^MLCT-driven electron transfer. Using actinometry to determine the photon flux of the experimental setup (Figure S14) together with the known absorption coefficient of MV^•+^, the reaction quantum yield was determined to be 2.4 × 10^–4^. Although the quantum yield appears to be relatively low, it cannot arise from diffusion-controlled ET: even at the diffusion limit of 6.5 × 10^9^ M^–1^ s^–1^ in water^99^ at a concentration of 10 mM MV^2+^, a maximum quantum yield of only 1.8 × 10^–4^ would be expected if every elementary step following electron transfer proceeded with 100% efficiency.^100,101^ In practice, additional processes such as cage escape, which often exhibit efficiencies of only 10%, would significantly reduce the observable quantum yield.

To test the role of micelles, we compared SDS with neutral and cationic micelles. Neutral micelles were expected to show little interaction with MV^2+^, while positively charged micelles should significantly suppress association of the dicationic photosensitizer and MV^2+^. In both cases, only a negligible signal of MV^•+^ was detected over prolonged irradiation, demonstrating that electron transfer event slows down in the absence of favorable electrostatic preassociation. The negligibly small residual signal can be attributed to diffusion-controlled reactivity, due to the strong absorptivity of MV^•+^ at 395 nm (ε = 42 000 M^–1^ cm^–1^),^102^ allowing us to detect traces of product formation.

To further support the conclusion that the observed reactivity originates from a higher-energy MLCT excited state, transient absorption quenching experiments were performed on [Fe­(bpy*N*py)_2_]^2+^ in 11 mM micellar solution, probing at 720 nm following 560 nm excitation in the presence of up to 1 M MV^2+^. No formation of MV^•+^ was detected, consistent with the low reaction quantum yield and the sensitivity limits of transient absorption spectroscopy, which typically requires electron-transfer efficiencies of about 10%, whereas continuous-wave experiments can detect substantially less efficient processes because of the much higher total photon flux. In addition, no excited-state lifetime quenching was observed, even at MV^2+^ concentrations as high as 1 M, indicating the absence of diffusion-based reactivity (Figure S19).

However, NMR titration experiments (Figure 4E, inset) showed that preassociation is already largely established at approximately 12.5 mM MV^2+^. When the transient absorption experiments were repeated in this concentration regime, a subtle but reproducible decrease in signal amplitude was observed (Figure 4E), consistent with static quenching due to photoinduced electron transfer within the preassociated complex.^103^ No further amplitude change was observed upon increasing the MV^2+^ concentration to 50 mM, indicating that the equilibrium had already shifted strongly toward the preassociated species (Figure 4E), in agreement with the NMR titration results. Correspondingly, smaller but systematic amplitude decreases were observed at 1 and 5 mM MV^2+^ (Figure S20), consistent with progressively increasing preassociation with increasing quencher concentration.

Viewed in the aggregate, these observations support a consistent picture in which ultrafast electron transfer originates from a higher-energy ^3^MLCT state prior to relaxation to a lower-lying MC state.

## Conclusion and Outlook

In this work we developed a modular Fe^II^ pyridinium-carbene platform that retains strong visible-light absorption while reinforcing strong ligand field relative to conventional NHC systems. Ultrafast variable-temperature transient absorption spectroscopy allowed comprehensive mapping of the excited-state landscape, including assignment of the spin-state manifold and determination of the zero-point energy between the lowest energy MC state and ground state via Marcus analysis. The modularity across both the bipyridine and pyridinium rings provides a versatile, tunable blueprint that should be adaptable to other first-row transition metal complexes.

Harnessing these energetic insights afforded thermodynamic control over photoredox reactivity, while strategic preassociation between photocatalyst and substrate imposed kinetic control. This enabled us to design a proof-of-concept electron transfer reaction from and something other than the compound’s lowest-energy excited state, specifically a ^3^MLCT state prior to energy loss, challenging the long-standing assumption that only the lowest-energy state of a given spin multiplicity can drive photochemistry.^104^ This approach is especially compelling for first-row transition-metal complexes, as up to 1–1.5 eV of excitation energy is lost before relaxation to the lowest excited state that can engage in productive chemistry.^105^ Conceptually, we believe this work points to a novel design principle for inorganic photochemistry. Rather than tuning the energy and lifetime of the lowest excited state – as has been the central strategy for decades – future efforts could instead focus on extending the lifetimes of higher-lying excited states into the hundreds of picoseconds-to-nanoseconds regime.^106,107^ If achieved, this would enable diffusion-controlled bimolecular photochemistry to occur prior to significant energy dissipation and open new avenues for first-row transition-metal-based light-to-energy conversion strategies.

## Supplementary Material



## Notes

General procedures and equipment details, as well as photophysical and photochemical data supporting conclusions presented in the paper (PDF)
