# Supplementary material for: Kinetics vs Thermodynamics: Engineering Photoredox Reactivity from an Upper Excited State of FeII
Source: J Am Chem Soc. 2026 Jun 4;148(23):23879–90. doi: 10.1021/jacs.6c02681 (PMC13281511; doi:10.1021/jacs.6c02681)
Supplement: Supplementary file 1 [file ja6c02681_si_001.pdf]

Supporting Information

Kinetics vs Thermodynamics: Engineering Photoredox Reactivity from an Upper Excited State of Fe^{II}

Atanu Ghosh[†], Björn Pfund[†], Jonathan T. Yarranton, Yi-Jyun Lien, James K. McCusker*

† these authors contributed equally

Department of Chemistry, Michigan State University, East Lansing, MI 48824, USA.

Corresponding author: jkm@chemistry.msu.edu

1. **General Information/Experimental/Methods**

Commercial reagents were used without further purification unless otherwise indicated. The solvents and reagents used for optical characterization and preparative irradiation experiments were obtained commercially in high purity and were used without further purification.
^{1}H and ^{13}C NMR spectra were recorded on a 500 MHz Agilent DDR2 spectrometer located at the Max T. Roger NMR facility, Michigan State University. NMR spectra were processed using Mnova, and the peaks were internally referenced to the residual solvent signal.[1] ESI-MS spectra were collected on a Waters G2-X2 QToF mass analyzer located at the Mass Spectrometry and Metabolomics Core Facility at Michigan State University. Elemental analysis for C, H and N was collected on a Perkin Elmer CHN Elemental Analyzer Model 2400 using the recrystallized and dried sample. UV-Vis absorption spectra were recorded using a Cary 50 from Varian with a 1 cm quartz glass cuvette.

Synthetic procedures and characterization data for all prepared compounds can be found in Sections 2 and 3 of the supplementary material.

Electrochemistry

Cyclic voltammetry (CV) measurements were conducted using a CH instrument model CH1620D electrochemical workstation in an argon filled glove box. The electrochemical setup consisted of a pseudo-reference electrode, a platinum disc working electrode, and a platinum wire as the counter electrode. CV experiments were performed at a sweep rate of 100 mVs^{-1} in deaerated acetonitrile solutions containing 0.1 M tetra-*n*-butylammonium hexafluorophosphate ($TBAPF_6$) as the supporting electrolyte. All voltammograms were referenced externally to the Fc/Fc^+ couple.

UV-Vis absorption spectro-electrochemistry was conducted in an argon-filled glove box using a Pine Research Instruments electrochemical cell. The cell was filled with 0.1 M $TBAPF_6$ solution in dry acetonitrile, and the 1 cm × 1 cm space at the top of the cell held the Ag reference electrode, while the Pt electrode serving as the working and counter electrode was placed in the 1.7 mm × 10 mm path length at the bottom of the cell. The UV-Vis absorption spectra were monitored using a S1420 CCD spectrometer, and the sample concentration was adjusted to achieve a maximum absorbance value of 0.6 ± 0.1. UV-Vis spectra of the initial compound were obtained before the onset of bulk electrolysis at an overpotential of 0.10 – 0.20 V relative to the oxidation/reduction potential of the compound.

Time Resolved UV-Vis Absorption Spectroscopy for Excited State Dynamics

Ultrafast transient absorption experiments were measured on a home-built laser system. See details in previous publications.[2,3,5] In short, a commercial Ti:sapphire oscillator (pumped by a 5.0 W diode laser) produces a 300 mW (76 MHz) mode-locked beam, which was used to seed a Ti:sapphire regenerative amplifier. The amplifier output (800 nm, 1 kHz) was split 70:30 between the pump and probe beams. The different pump wavelengths were generated from an optical parametric amplifier (TOPAS, Light Conversion). The probe beam was generated from a white light continuum using either a sapphire crystal or CaF_2. If not specified, data were collected at 20 °C in a 2 mm quartz cuvette with ~4 μJ pump power. UV-Vis spectra of each sample were collected after the experiment to ensure the sample had not decomposed. Variable-temperature measurements were conducted through the incorporation of an optical Dewar (Janis Research

SuperTran-VT 100) into the sample region.[2] All data were processed using IGOR Pro software by wavemetrics, and kinetic fitting were done using a biexponential fit function.

For full-spectrum data acquisition, the white light probe was directed to a spectrometer (CAM-VIS-2 , Ultrafast Systems, grating #4). For all measurements, the probe polarization was set to 54.7° with respect to the pump polarization to eliminate any anisotropy. The data were chirp-corrected using commercially available Surface Explorer software, provided by Ultrafast Systems. SVD analysis of the full spectra data was performed using open-source Glotaran software.[4]

2. Synthesis of $[Fe(bpy\textit{N}py)_2]^{2+}$ Complex.

[1,2':6',2''-terpyridin]-1-ium hexafluorophosphate ([bpy*N*PyH]PF_6) was prepared using a modified literature procedures.[6] Bipyridine mono *N*-oxide (2.00 g, 11.61 mmol) was dissolved in MeCN (50 mL), and pyridine (5.60 mL, 69.68 mmol) was added dropwise to the stirred solution. The solution was cooled to 0 °C, and trifluoromethanesulfonic anhydride (2.41 mL, 17.42 mmol) was added dropwise over 30 mins. The orange solution was stirred at 0 °C for 30 minutes, then allowed to warm to room temperature and stirred overnight. Solvent was removed under reduced pressure, and the remaining solid was dissolved in MeOH (20 mL). To this orange solution, NH_4PF_6 (5.68 g, 34.83 mmol) was added. The precipitate was filtered and washed with H_2O and MeOH. The crude white powder was used without any further purification. Yield: 3.21 g, 73%.

^1H NMR (CD_3CN, 298K, 500 MHz) δ [ppm]: 9.48 – 9.46 (m, 2H), 8.80 – 8.75 (m, 3H), 8.54 (dd, J = 8.1, 1.0 Hz, 1H), 8.38 (t, J = 8.0 Hz, 1H), 8.29 (t, J = 7.1 Hz, 2H), 8.00 (td, J = 8.0, 2.3 Hz, 2H), 7.52 (dd, J = 7.6, 4.7 Hz, 1H).

$[Fe(bpy\textit{N}Py)_2](PF_6)_2$ was synthesized using a modified literature procedure.[7] $FeBr_2$ (60 mg, 0.28 mmol) and [bpy*N*Py]PF_6 (0.25 g, 0.66 mmol) were suspended in EtOH (5 mL) under anaerobic conditions in a sealed pressure tube. Triethylamine (1.55 mL, 11.13 mmol) was added, resulting in the immediate formation of a grayish-green cloudy solution. The mixture was heated to 100 °C (under pressure), resulting in a blue-purple color, and stirred for 16 hours. The reaction mixture was allowed to cool to 25 °C and was added to a saturated $KPF_{6(aq)}$ (50 mL) aqueous solution forming a blue-purple solid. The crude blue-purple precipitate was filtered and washed with H_2O and MeOH. The crude was purified by column chromatography with silica gel, eluting with a gradient mixture starting from MeCN, followed by a MeCN/H_2O (v/v = 1/1) mixture, and ending with MeCN / H_2O / Sat. $KNO_{3(aq)}$ (v/v/v = 5/4/1), affording a purple powder. Yield: 0.17 g, 76%.

^1H NMR (acetone-d_6, 298 K, 500 MHz) δ [ppm]: 9.83 (d, J = 6.9 Hz, 2H), 9.14 (d, J = 8.0 Hz, 2H), 9.07 (d, J = 8.4 Hz, 2H), 8.86 (dt, J = 8.1, 1.1 Hz, 2H), 8.76 (t, J = 8.2 Hz, 2H), 8.13 (td, J = 7.8, 1.5 Hz, 2H), 7.59 (dt, J = 5.4, 1.2 Hz, 2H), 7.39 – 7.29 (m, 6H), 6.61 (d, J = 7.9 Hz, 2H).

^{13}C NMR (CD_3CN, 298K, 126 MHz) δ [ppm]: 227.76, 159.21, 158.48, 154.90, 151.41, 140.90, 139.18, 137.51, 136.69, 136.08, 127.82, 124.67, 122.40, 119.35, 114.46.

HRMS (ESI-TOF) m/z: $[M-2(PF_6)]^{2+}$ calc'd for $C_{30}H_{22}N_6Fe$: 261.0628, obs. 261.0688.

Elemental analysis: Anal. Calcd. for $C_{30}H_{22}N_6Fe$: C, 44.30; H, 2.70; N, 10.34; found: C, 43.53; H, 2.72; N, 10.01.

3. NMR and High Resolution MS Spectra of Synthesized Compounds

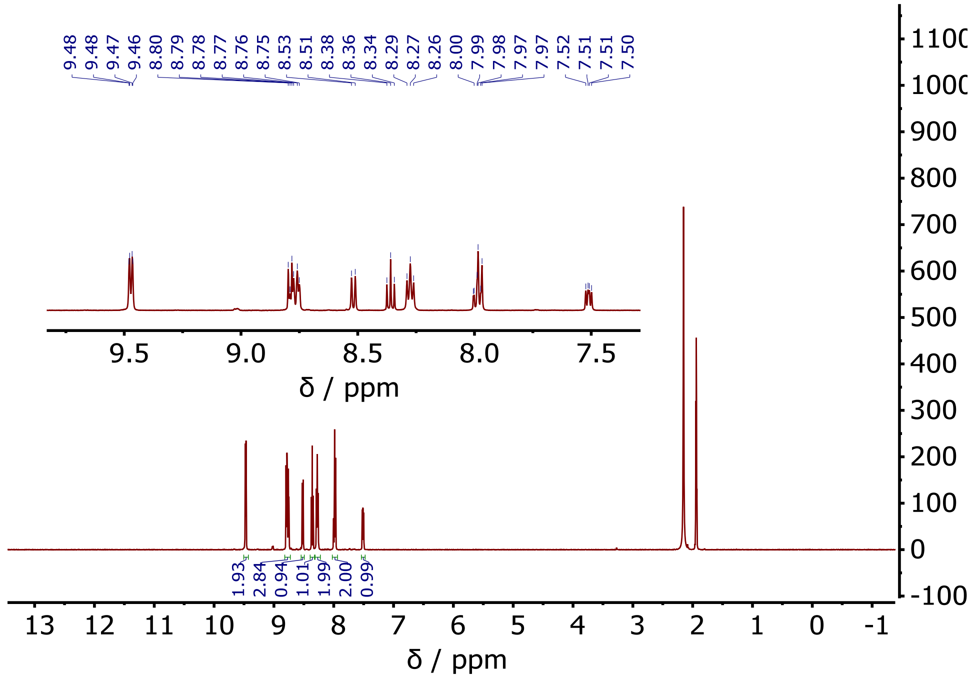

Figure S1: ^{1}H-NMR spectrum of [bpy*N*PyH]PF_6 in CD_3CN at 298 K.

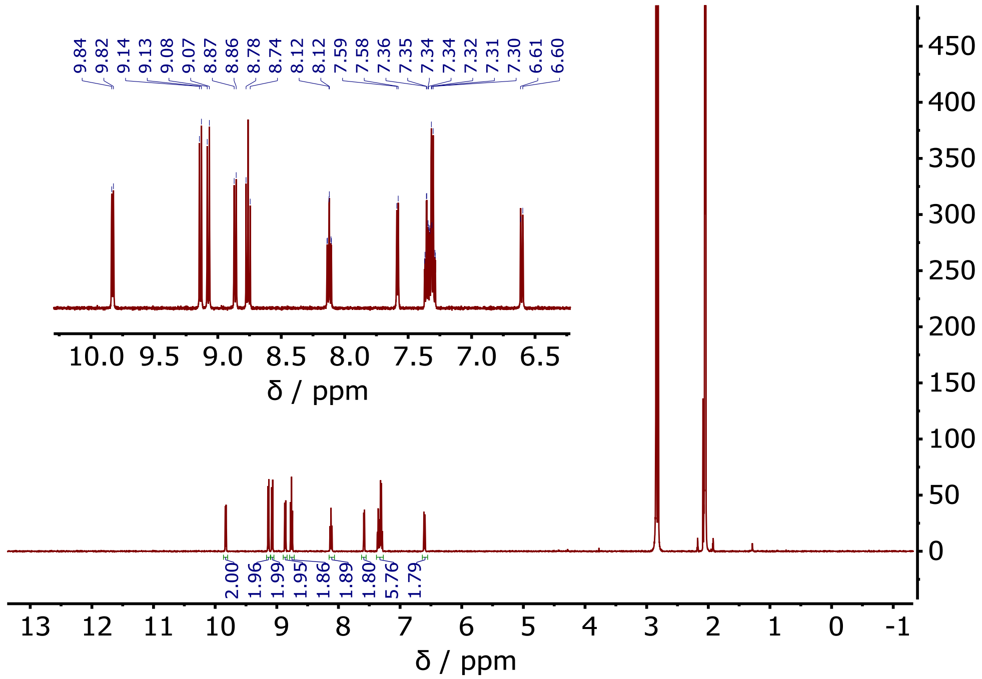

Figure S2: ^{1}H-NMR spectrum of [Fe(bpy*N*py)$_2$]$(PF_6)_2$ in d_6-acetone at 298 K.

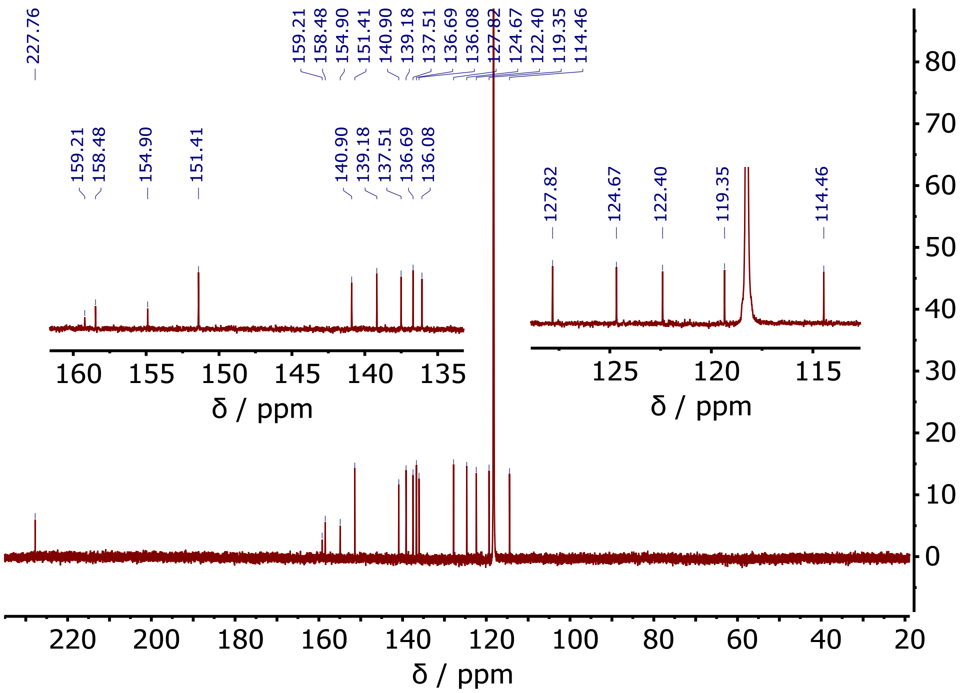

Figure S3: ^{13}C-NMR spectrum of [Fe(bpy*N*py)$_2$](PF$_6$)$_2$ in CD$_3$CN at 298 K.

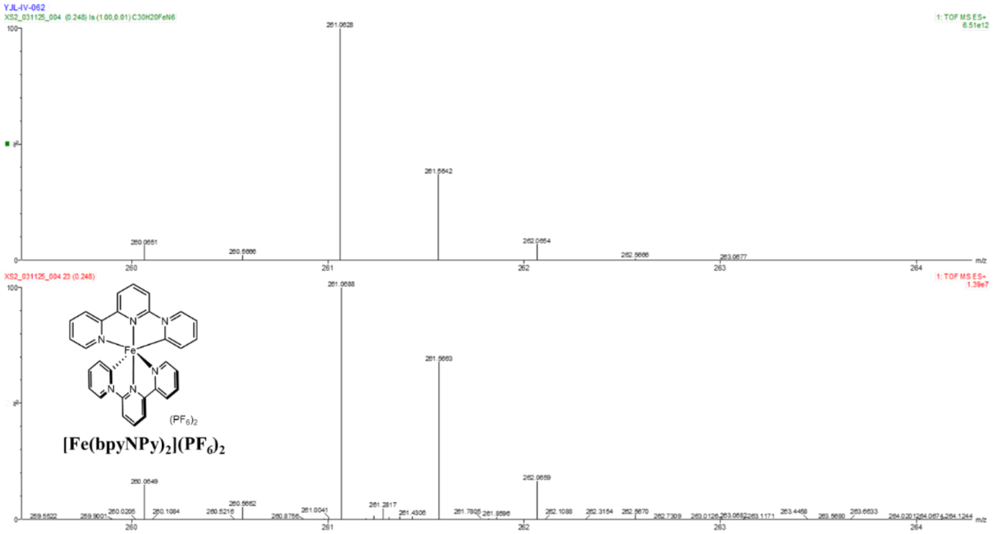

Figure S4: Calculated (top) and experimental (bottom) high resolution electrospray ionization mass spectrum ESI-MS of [Fe(bpy*N*py)$_2$](PF$_6$)$_2$.

4. Spectroelectrochemistry

Spectroelectrochemistry has proven to be both useful and, at times, misleading for assignments of excited states.[8, 9] Therefore, we employed multiple approaches to analyze and interpret our results, as discussed in the main manuscript and further elaborated in the following section of the supplementary information. To simulate the spectrum of the MLCT state, spectroelectrochemical experiments were conducted on $[Fe(bpy\textit{N}py)_2](PF_6)_2$.

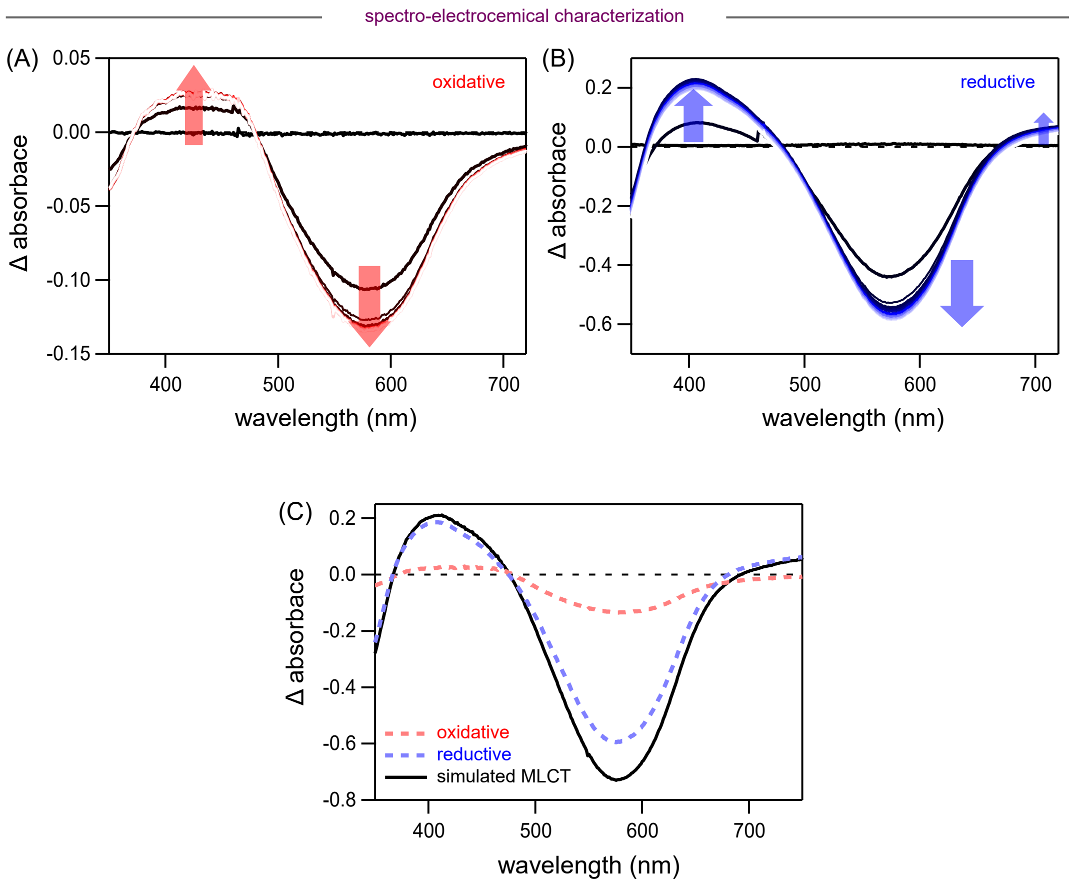

Figure S5: Spectroelectrochemical results of $[Fe(bpy\textit{N}py)_2](PF_6)_2$ in 0.1 M $TBAPF_6$ solution in acetonitrile. (A) oxidative spectra collected at 0.55 V applied potential (vs Fc/Fc^+) as a function of time (B) reductive spectra collected at −1.60 V applied potential (vs Fc/Fc^+) as a function of time and (C) predicted MLCT spectrum (solid black trace) is calculated by addition of oxidative (dotted, red) and reductive (dotted, blue) spectra. For spectroelectrochemistry experiments, the absorption of the sample solution was 0.4-0.6 at the absorption maximum.

The oxidative spectra exhibit a broad, positive absorptive signal near 430 nm, with the lower energy region dominated by bleach. The positive absorption can be assigned to the LMCT absorption of Fe(III). Whereas the reductive spectra show two positive absorptions, one in the 400 nm region and the other one beyond 660 nm, characteristic of reduced bipyridine absorption bands (here, $bpy\textit{N}py^{\bullet-}$). We suspect that the pronounced bleach in reductive spectroelectrochemistry originates from the irreversible nature of the reduction process.

5. Additional Transient Absorption Studies

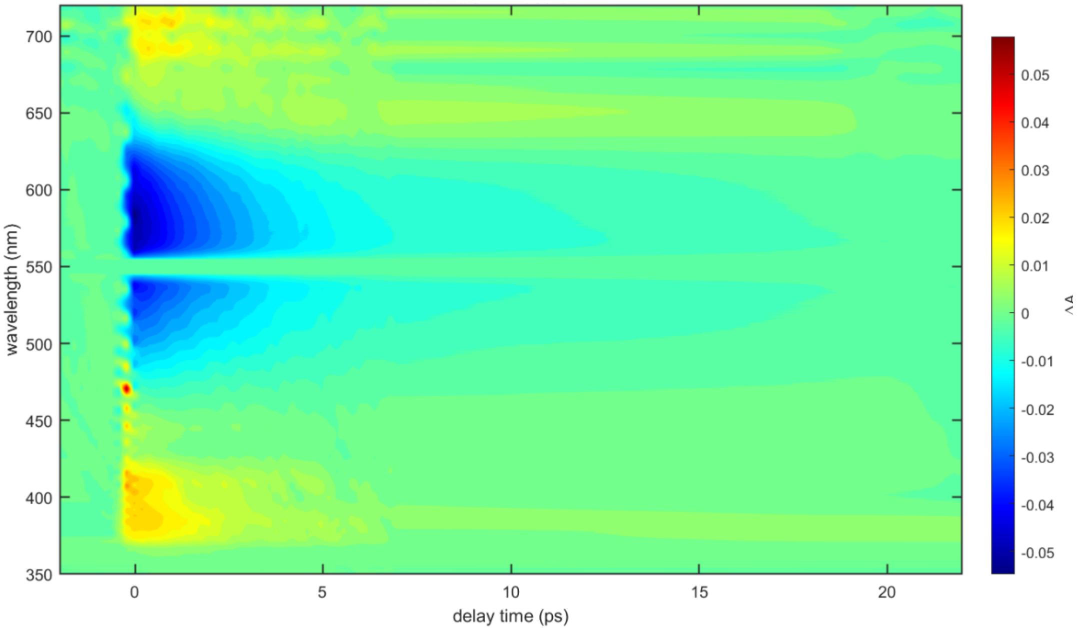

Figure S6: Chirp corrected transient absorption full spectra map of [Fe(bpy*N*py)$_2$](PF$_6$)$_2$, collected in acetonitrile solution at 20 °C following 550 nm photoexcitation. Data in the region of 550 ± 10 nm are compromised due to pump scattering. The colormap shown on the right represents the amplitude of the change in absorbance.

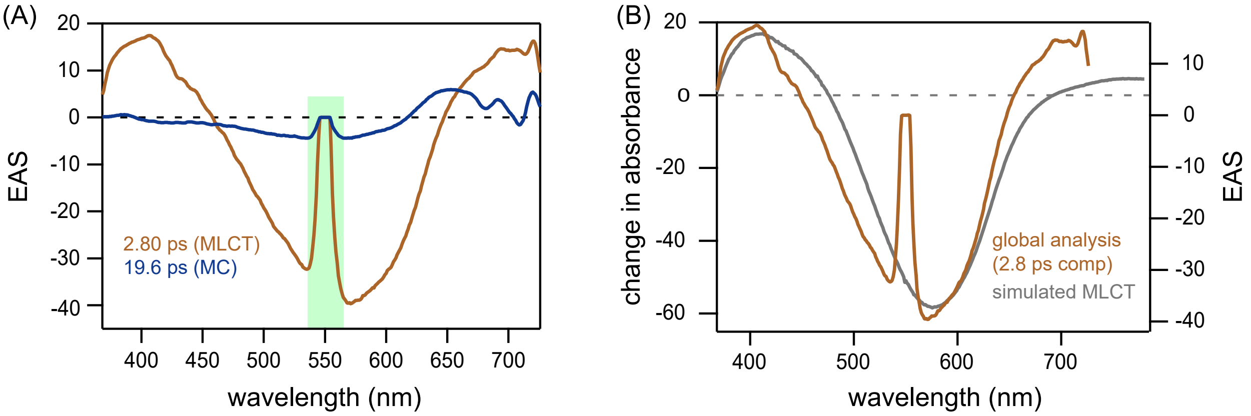

Figure S7: (A) Evolution-associated spectra (EAS) obtained from the global analyses of the transient absorption data. A two-component sequential excited-state evolution model was employed to reproduce the experimental data satisfactorily. The 2.8 ps time component is assigned to the ^{1}MLCT→MC conversion, as the spectra feature closely matches the simulated MLCT spectrum (see panel B). The ~20 ps component is assigned to the ground state recovery (MC→GS) process. See main text and discussion for further details. The shaded region around 550 nm is compromised due to pump scattering.

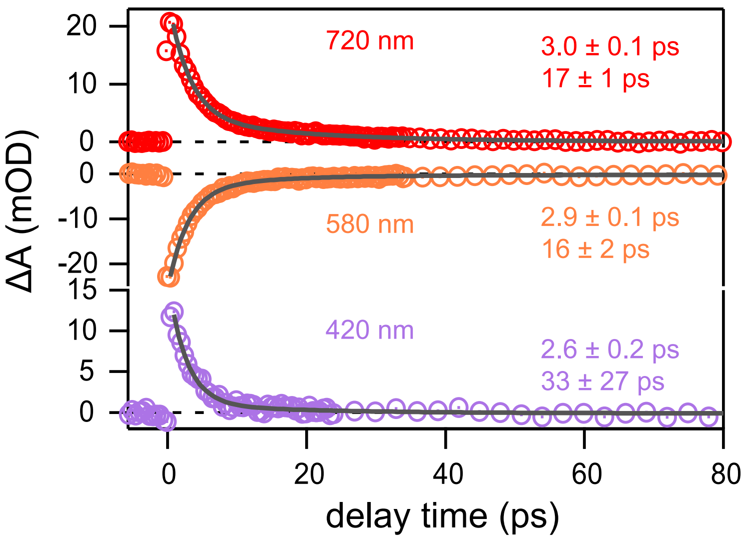

Figure S8: Transient absorption kinetic traces of $[Fe(bpyNpy)_2](PF_6)_2$ collected at 720 nm, 580 nm, and 420 nm in acetonitrile solution following 550 nm photoexcitation. The solid grey line at each trace represents the bi-exponential fit. In all cases, a minimum of two exponential components was required to fit the experimental kinetic traces satisfactorily. Their resultant time constants are provided in the plot and are color-coded.

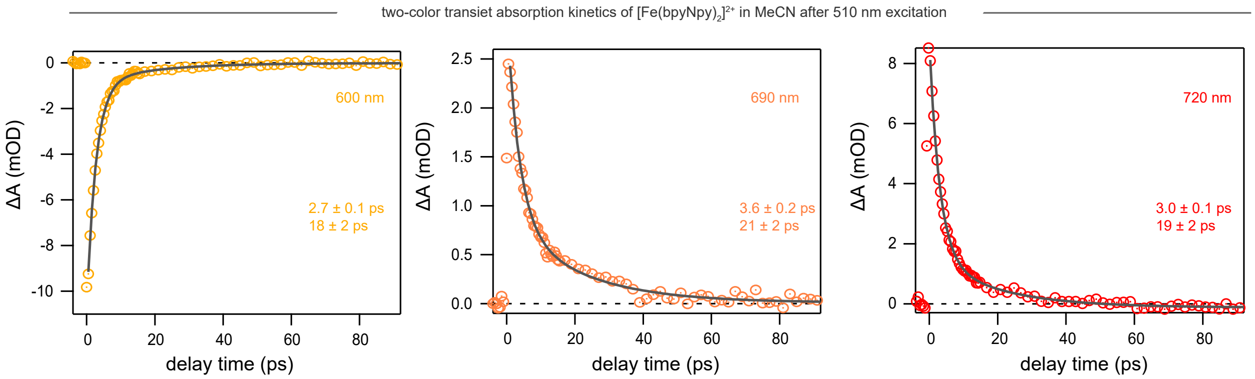

Figure S9: Transient absorption kinetic traces of $[Fe(bpyNpy)_2](PF_6)_2$ collected at 600 nm, 690 nm, and 720 nm in acetonitrile solution following 510 nm photoexcitation. The solid grey line at each trace represents the bi-exponential fit. In all cases, a minimum of two exponential components was required to fit the experimental kinetic traces satisfactorily. Their resultant time constants are provided in the plot and are color-coded.

Thermalized Metal-centered state. Similarities in time constants for the ground state recovery process between the 550 nm and 510 nm excitations suggest that the ground state recovery proceeds from a thermalized MC state, an important consideration for variable-temperature measurements discussed in the next section.

6. Variable-Temperature Transient Absorption Studies

Variable-temperature transient absorption (VT-TA) spectroscopy has proven to be a powerful tool for disentangling nonradiative decay processes in transition metal complexes.[2, 10, 11] Recently, we demonstrated that spin-state-sensitive information can be extracted from VT-TA experiments when analyzed through nonradiative decay theories, particularly transition-state theory and semi-classical Marcus theory. Although, in principle, both ^{1}MLCT→MC and MC→GS processes can be examined using VT-TA, it is essential to note that the decay process under investigation must originate from a thermalized state; otherwise, temperature has no meaningful interpretation. Therefore, in this study, we focus solely on the VT-TA results for the MC→GS ground state recovery process as it starts from the thermalized MC state (see Figure S9 and associated discussion).

For the VT-TA experiments on the [Fe(bpy*N*py)$_2$](PF$_6$)$_2$ complex, a probe wavelength of 660 nm was selected as the MC exhibits a strong absorption at this wavelength. Transient absorption kinetic traces were recorded at 660 nm following 550 nm photoexcitation over a temperature range of 273 K to 298 K. If a complex possesses a measurable activation barrier, this temperature window should be sufficient to reveal it, as differences in lifetime will become evident in these measurements. For instance, we recently reported VT-TA kinetics of a series of d^6 low-spin Co(III) complexes, where the kinetics were found to operate in the Marcus inverted region. In the case of [Co(pyrro-bpy)$_3$](PF$_6$)$_3$ complex (where pyrro-bpy is 4,4'-di-pyrrolodine-2,2'-bipyridine), the activation barrier for the ground state recovery process was determined to be 850 ± 50 cm^{-1}. From the VT-TA traces, the lifetimes at 298 K and 273 K are 440 ± 20 ps and 670 ± 25 ps, respectively, representing a 52% increase.[10]

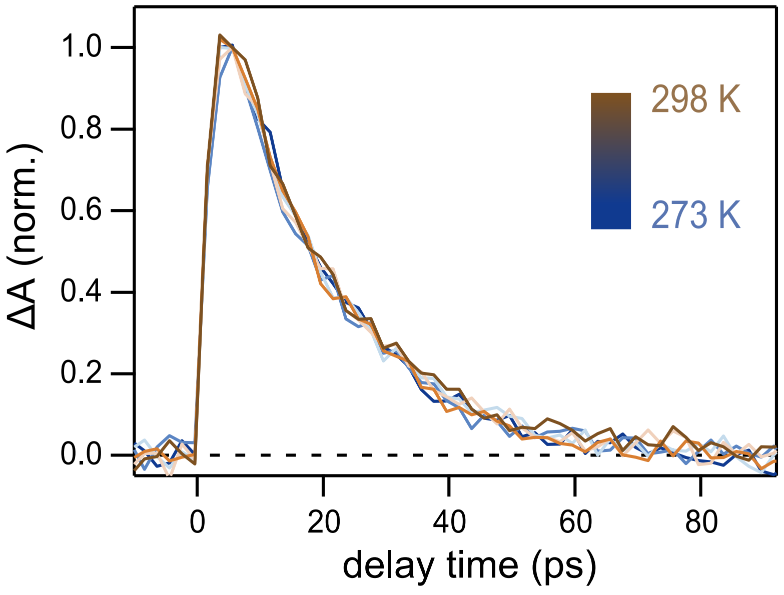

Figure S10: Ground state recovery kinetics of [Fe(bpy*N*py)$_2$](PF$_6$)$_2$ as a function of temperature (from 273 K to 298 K with 5 K interval). The kinetic data were collected at 660 nm in fluid acetonitrile solution following 550 nm photoexcitation.

From the VT-TA data of $[Fe(bpy\textit{N}py)_2](PF_6)_2$, it became evident that the lifetime remains essentially unchanged within this temperature range, indicating the presence of only a minimal barrier (< thermal energy). Such a negligible barrier is consistent with the observed 20 ps ground state recovery lifetime, which is notably faster than that of other Fe(II) and Co(III) polypyridyl complexes. Further details on the Arrhenius analysis can be found in the main document.

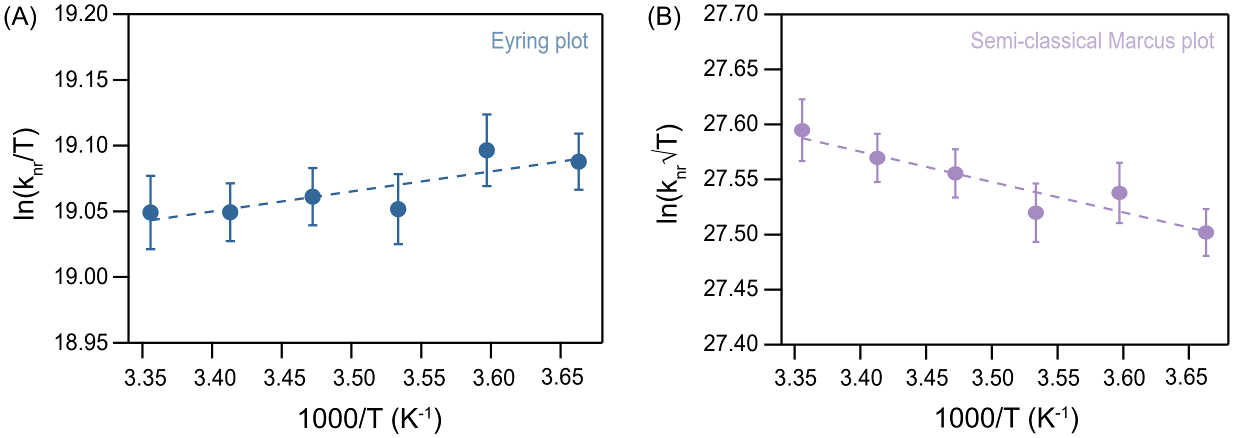

Figure S11: Eyring and semi-classical Marcus plots, obtained from the variable-temperature ground state recovery measurements on $[Fe(bpy\textit{N}py)_2](PF_6)_2$. Activation entropy and activation enthalpy were obtained from the Eyring analyses, and reorganization energy, electronic coupling, and driving force values were estimated from the semi-classical Marcus analysis. See main text and discussion for further details.

To gain further insights into the thermodynamics of the ground state recovery process, transition-state theory and semi-classical Marcus analyses were performed, with detailed discussion presented in the main text. The corresponding plots and fittings are shown in Figure S11.

$$k_{nr} = \kappa \frac{k_B T}{h} exp\left\{\frac{\Delta S^\ddagger}{k_B}\right\} exp\left\{-\frac{\Delta H^\ddagger}{k_B T}\right\} \quad \text{(Eq. S1)}$$

Eq. S1 presents the Eyring model where k_{nr} is the nonradiative decay constant, k_B is Boltzmann constant, T is absolute temperature, and κ is transmission coefficient – often assumed to be 1 as a simplification. Fitting the VT-TA data using the linearized Eyring equation provides $\Delta H^\ddagger$ and $\Delta S^\ddagger$ from the slope and intercept, respectively. An intriguing outcome from the transition-state theory analysis using the Eyring model (Eq. S1) is the positive slope observed in the $\ln(k_{nr}/T)$ vs. 1000/T plot, which is discussed in the main manuscript.

<u>Additional Details on the Semi-Classical Marcus Analysis</u>

As described earlier, the semi-classical Marcus equation contains three unknowns (ΔG_0, λ, and H_{ab}), while only two quantities, k_{nr} and T, are experimentally accessible. As a consequence, it is not possible to determine all three unknowns precisely. However, in this case, since the ground state recovery process operates in the barrierless region, the exponential activation term vanishes, leading to a condition $\Delta G_0 \approx \lambda$. This unique photophysical behavior allows us to estimate both the

zero-point energy difference and the electronic coupling term (H_{ab}) between the excited and ground state.
To strengthen our assignment of the spin-state for the MC state, we considered a range of plausible reorganization energies and calculated the corresponding electronic coupling matrix elements. For reference, we previously reported that a series of tris-bipyridyl Co(III) complexes exhibit a reorganization energy of approximately ~5600 cm^{-1} for the $^{3}MC \rightarrow GS$ conversion, whereas Carey, McCusker, and co-workers estimated a reorganization energy of 14000 cm^{-1} for the $^{5}MC \rightarrow GS$ process in a bis-terpyridyl Fe(II) complex.[2, 10] Using the VT-TA results of $[Fe(bpy\textit{N}py)_2](PF_6)_2$, electronic coupling values were extracted from the intercept of the semi-classical Marcus plot, employing both reorganization energy estimates. The resulting H_{ab} values were 21.2 cm^{-1} (for λ = 5600 cm^{-1}) and 26.7 cm^{-1} (for λ = 14000 cm^{-1}). Notably, these magnitudes of H_{ab} are significantly larger than those typically observed for Fe(II) polypyridyl complexes (3 – 5 cm^{-1}), where the coupling is between the $^{5}T_2$ and $^{1}A_1$ states ($\Delta S = 2$ process). Instead, the obtained H_{ab} values fall within the range reported for Co(III) polypyridyl complexes, where the ground state recovery proceeds from a ^{3}MC state ($\Delta S = 1$ process). Therefore, our VT-TA data strongly support the conclusion that the lowest energy MC state in this case is the ^{3}MC. We recently reported photoredox reactivity originating from the ^{3}MC excited state of Co(III) complexes. [2, 10] Therefore, it was tempting to explore analogous reactivity in this system as well. However, to assess the feasibility of such reactivity, it is first necessary to estimate the zero-point energy of the excited state.

Possible Range for Reorganization Energies (λ) and Estimation of Zero-point Energy.

As we have established that the lowest energy state in $[Fe(bpy\textit{N}py)_2](PF_6)_2$ is the ^{3}MC state, which is represented by the $(t_{2g})^5(e_g^*)^1$ electronic configuration, one can anticipate reorganization energy values approximately half of that has been estimated for the Fe(II) bis-terpyridine $[Fe(tpy)_2](PF_6)_2$ complex. In the case of Fe(II), the structural change accompanying the ground state recovery process is nearly double due to the $^{5}MC \rightarrow GS$ transition, where ^{5}MC and ^{1}GS are represented by $(t_{2g})^4(e_g^*)^2$ and $(t_{2g})^6(e_g^*)^0$ electronic configurations, respectively. Therefore, a reorganization energy of about 7000 cm^{-1} served as a reasonable initial approximation. However, we expect a somewhat higher reorganization energy for this Fe-carbene-complex compared to polypyridyl analogs, due to the presence of Fe–C bonds, which have a higher force constant than Fe–N bonds. Consequently, the reorganization energy for the ground-state recovery process in $[Fe(bpy\textit{N}py)_2](PF_6)_2$ is likely to be slightly more than 7000 cm^{-1}. Therefore, within a reasonable approximation, we considered 5000 – 9000 cm^{-1} as a possible range for reorganization energy associated with the ground state recovery process. Based on the "barrierless" dynamics, the zero-point energy was estimated to be 5000 – 9000 cm^{-1} from Marcus theory.

Error propagation for Arrhenius, Eyring and Marcus parameters.

The reported uncertainties in the Arrhenius activation barrier, activation entropy and enthalpy, reorganization energy, and electronic coupling were obtained by propagating the errors associated with the GSR lifetimes. The uncertainties in the GSR lifetimes were determined from the standard deviations of multiple independent measurements. Each dataset was evaluated for internal consistency prior to averaging, and the resulting mean lifetimes and corresponding standard deviations were used for the error analysis. Although mathematically rigorous error propagation

is one approach to estimating uncertainties, it often underestimates the true error bars. We therefore evaluated the physical parameters using the two extreme values defined by the upper and lower bounds of the fitted slopes and intercepts. The standard deviation of the resulting parameter range was taken as the uncertainty for the Arrhenius, Eyring, and Marcus parameters reported here. For example, the intercept of the Eyring plot was found to be 18.5 ± 0.32. Two extremes of the intercept, 18.18 and 18.82, result in activation entropies of −3.88 $cm^{-1}K^{-1}$ and −3.43 $cm^{-1}K^{-1,}$ respectively. Therefore, the activation entropy reported is −3.65 ± 0.22 $cm^{-1}K^{-1}$.

Photoredox Catalysis from Higher Excited States

Pre-association of MV^{2+} with SDS Micelle

To enable picosecond reactivity, pre-association between the photocatalyst and the substrate is required. To promote such interactions, we employed a micellar superstructure in which the water-insoluble complex [Fe(bpy*N*py)$_2$](PF$_6$) is incorporated into the hydrophobic core of anionic SDS micelles. The water-soluble substrate, methyl viologen (MV^{2+}), can associate with the negatively charged micellar surface through electrostatic interactions. While the incorporation of [Fe(bpy*N*py)$_2$](PF$_6$) into the micelles is evident from its solubility in the aqueous micellar solution, the interaction of MV^{2+} with SDS micelles is less straightforward. To probe this association, UV-Vis absorption spectra of MV^{2+} were recorded in the presence and absence of SDS micelles, revealing distinct spectral shifts indicative of pre-association (Figure S12). In contrast, for neutral (Triton X-100) and cationic (acetylpyridinium chloride) surfactants, the MV^{2+} absorption overlapped with the micellar background, preventing clear identification of such interactions.

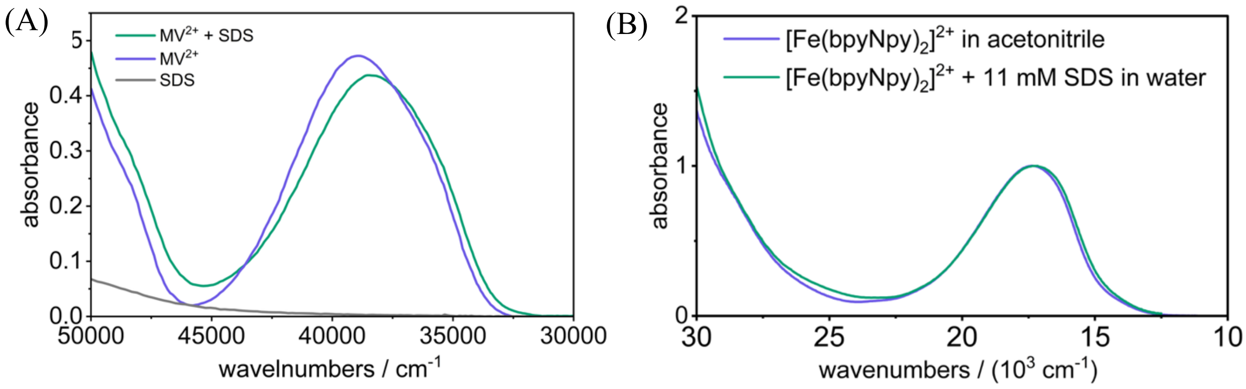

Figure S12: (A) UV-Vis absorption spectra of 2 × 10^{-5} M MV^{2+} in water (purple line) in the presence of 8.3 mM SDS micelles (green line), and of 8.3 mM SDS in water alone. The spectral changes indicate aggregation of MV^{2+} with the anionic micelles, consistent with the formation of a pre-associated complex. (B) UV-Vis absorption spectra of $[Fe(bpyNpy)_2]^{2+}$ in acetonitrile (purple line) and in aqueous solution in the presence of 11 mM SDS micelles (green line). The small changes in the absorption profile likely reflect differences in solvent polarity between acetonitrile and the micellar environment.

Choosing the Concentration for Surfactant

To ensure comparable conditions among the different micelle systems, the surfactant concentration was chosen so that each micelle contains one photocatalyst. This allows for efficient pre-association with MV^{2+} while preserving the solubility of $[Fe(bpy\textit{N}py)_2]^{2+}$.

The molar concentration of micelles ([micelles]) can be estimated using Eq. S2,

$$[\text{micelles}] = \frac{[S]_{\text{total}} - CMC}{N_{\text{agg}}} \qquad \text{(Eq. S2)}$$

where $[S]_{\text{total}}$ is the total surfactant concentration, *CMC* is the critical micelle concentration, and N_{agg} is the aggregation number.

In the photocatalytic experiments, a photocatalyst concentration of 80 μM $[Fe(bpy\textit{N}py)_2]^{2+}$ was used. Based on the critical micelle concentration (*CMC*) and aggregation number (N_{agg}) of the respective surfactants (Table S1), total surfactant concentrations of 11 mM for SDS, 12 mM for TX-100, and 9 mM for CPC were employed, ensuring comparable micellar systems across all experiments.

Table S1. Overview of key surfactant properties governing micelle formation, such as the critical micelle concentration (*CMC*), aggregation number (N_{agg}), and the concentration of the surfactant used for photocatalysis.

Surfactant	Critical Micelle Concentration (*CMC*) / mM	Aggregation Number (N_{agg})	Concentration of the Surfactant [a] /mM
Sodium Dodecyl Sulfate (SDS)	8.3[b]	36[c]	~11
Triton-X 100 (TX100)	0.224[d]	147[e]	~12
Cetylpyridinium Chloride (CPC)	0.95[f]	104[f]	~9

[a] Surfactant concentrations were estimated from Eq. S2 to match the micelle concentration corresponding to the photocatalyst concentration (80 μM). [b] Value obtained from reference.[12] [c] Value obtained from reference.[13] [d] Value obtained from reference.[14] [e] Value obtained from reference [15]. [f] Value obtained from reference [16].

Control Experiments for Excited-State Electron-Transfer Reactivity

In the photocatalytic experiments, standard conditions employed 100 mM TEOA, 11 mM SDS, 10 mM MV^{2+}, and 80 μM $[Fe(bpy\textit{N}py)_2]^{2+}$, using a 150 W xenon arc lamp with the output passed through a monochromator to isolate 560 ± 10 nm light and a long-pass filter to remove wavelengths below 480 nm. Control experiments were performed without TEOA, without $[Fe(bpy\textit{N}py)_2]^{2+}$, and without light irradiation, all of which resulted in the absence of $MV^{\bullet+}$ formation (Figure S13).

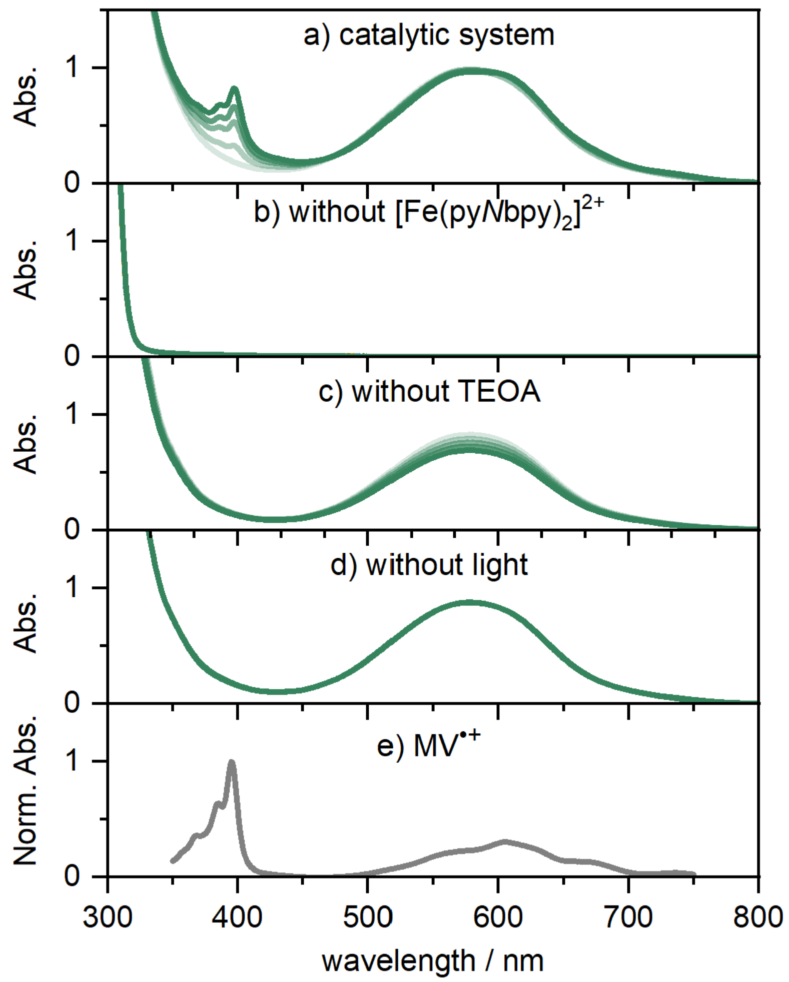

Figure S13: Control experiments probing electron-transfer reactivity from the higher-energy MLCT state of $[Fe(bpy\textit{N}py)_2]^{2+}$ to MV^{2+}. Standard conditions employed 100 mM TEOA, 11 mM SDS, 10 mM MV^{2+}, and 80 μM $[Fe(bpy\textit{N}py)_2]^{2+}$, using a 150 W xenon arc lamp with the output passed through a monochromator to isolate 560 ± 10 nm light and a long-pass filter to remove wavelengths below 480 nm. The corresponding experiment under these conditions is shown in (A) (identical to Figure 4D in the main manuscript). Control experiments (B) without $[Fe(bpy\textit{N}py)_2]^{2+}$, (C) without TEOA, and (D) without light irradiation were performed. For comparison, the UV-Vis difference absorption spectrum obtained upon electrochemical reduction of MV^{2+} to $MV^{\bullet+}$ at −1.1 V vs Fc/Fc^+ in acetonitrile is shown in (E).

Actinometry Experiments

The photon flux of the emission spectrometer equipped with a 150 W xenon arc lamp and a monochromator set to 580 ± 10 nm was determined by chemical actinometry using Reinecke's salt, following the literature procedure.[17]

In short, Reinecke's salt (0.015 M) was dissolved in 0.1 M aqueous KOH, and the pH was adjusted between 4 and 5 by the addition of concentrated sulfuric acid (98%). The resulting solution was irradiated for up to 21 minutes using the above-mentioned conditions. After irradiation, 0.1 mL of the solution was mixed with 0.3 mL of an aqueous solution containing $Fe(NO_3)_3$ (0.1 M) and HNO_3 (0.5 M), and subsequently diluted with water to yield a maximum absorbance of 0.55 OD at 460 nm (dilution factor = 72). The obtained absorptivity at 460 nm was then used to calculate the number of photons in Einstein (E) over time using Eq. S3:

$$E = \frac{(A_{460}^{t} - A_{460}^{0}) \times V}{\varepsilon_{460} \times d \times \Phi_{reac.}} \times f \times \frac{1}{1 - T_{580}} \qquad \text{(Eq. S3)}$$

where A^{t}_{460} is the absorbance at 460 nm after irradiation, A^{0}_{460} is the initial absorbance at 460 nm before irradiation, V is the volume of the sample solution (3.5 mL), ε_{460} is the molar absorption coefficient at 460 nm (3400 M^{-1} cm^{-1}), d is the path length of the cuvette (1 cm), $\Phi_{reac.}$ reaction quantum yield when using 580 nm light irradiation ($\Phi_{reac.}$ = 0.26),[17] f the dilution factor (f = 72), and T_{580} is the transmittance of Reinecke's salt at 580 nm (0.162), which corrects for the fraction of light that is not absorbed by the actinometer solution.
Plotting the number of absorbed photons versus irradiation time yielded a linear relationship, corresponding to a photon flux (φ_q) of 6.7 ± 0.1 × 10^{-8} E s^{-1}.

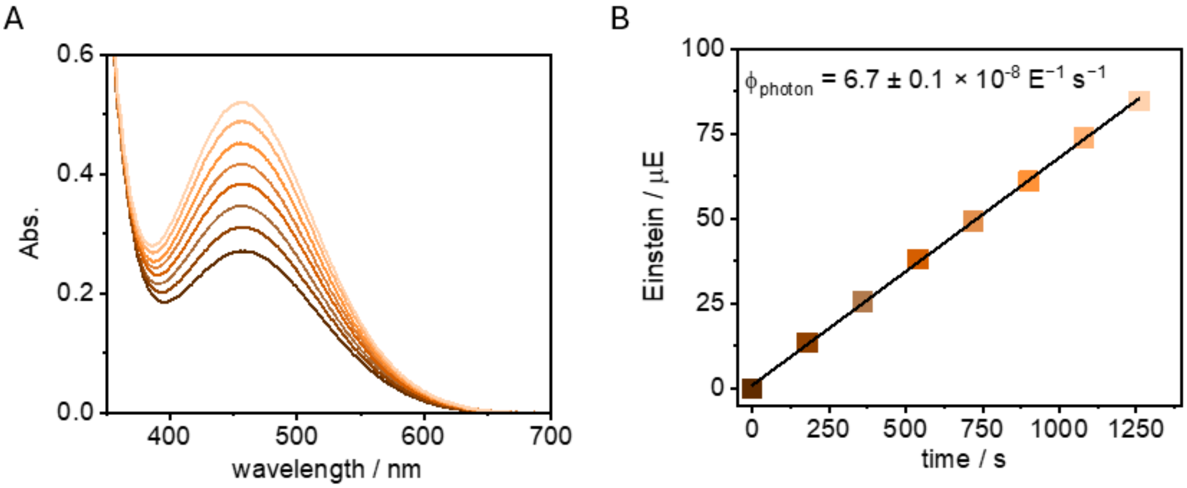

Figure S14: A. UV-Vis absorption spectra recorded at different time points during the photoirradiation of Reinecke's salt followed by quenching with $Fe(NO_3)_3$ in aqueous 0.5 M HNO_3, forming Fe(SCN), which exhibits a characteristic absorption band at 460 nm. B. Number of photons over time, calculated using Eq. S3 and the data from panel A. The color code corresponds to the different time points shown in panel A.

Reaction Quantum Yield

With the photon flux of the irradiation setup known, the reaction quantum yield of the MV^{2+} reduction ($\Phi_{reac.}$) was determined according to Eq. S4:

$$\Phi_{reac} = \frac{(A_{395}^{t} - A_{395}^{0}) \times V}{\varepsilon_{395} \times d} \times \frac{1}{\varphi_{photon} \times t} \quad \text{(Eq. S4)}$$

where A^0_{395} and A^t_{395} are the absorbance of $MV^{•+}$ at 395 nm before and after irradiation, , V is the volume of the sample solution (4 mL), ε_{395} is the molar absorption coefficient of $MV^{•+}$ at 395 nm (42 000 M^{-1} cm^{-1}), d is the path length of the cuvette (1 cm), φ_q is the photonflux of the light source ($6.7 \pm 0.1 \times 10^{-8}$ E s^{-1}), and t the irradiation time used in the photocatalytic experiment.

SDS Micelle

Using the photocatalysis results shown in Figure 4 of the main manuscript, a reaction quantum yield of 2.4×10^{-4} was obtained under standard conditions. Although the quantum yield seems relatively low, it cannot arise from diffusion-controlled ET, as even at the diffusion limit of 6.5×10^9 M^{-1} s^{-1} in water[18] at a concentration of 10 mM MV^{2+}, a maximum quantum yield of approximately 1.8×10^{-44} is expected, when every elementary step following electron transfer proceeded with 100% efficiency.

TX100 Micelle

Using the photocatalysis results shown in Figure 4 of the main manuscript, a reaction quantum yield of 2.4×10^{-5} was obtained under standard conditions using TX100 surfactant. However, this value is likely overestimated due to the formation of a decomposition product absorbing in the same spectral region, particularly since only minor spectral changes were observed compared to the SDS micellar system. Nevertheless, this finding suggests that the small amount of $MV^{•+}$ formation may arise from a diffusion-controlled process where a maximum theoretical quantum yield of approximately 1.8×10^{-4} can be expected.

CPC Micelle

Using the photocatalysis results shown in Figure 4 of the main manuscript using CPC surfactant, a reaction quantum yield of 1.4×10^{-5} was obtained under standard conditions. However, this value is likely overestimated due to the formation of a decomposition product absorbing in the same spectral region, particularly since only minor spectral changes were observed compared to the SDS micellar system. Nevertheless, this finding suggests that the small amount of $MV^{•+}$ formation may arise from a diffusion-controlled process where a maximum theoretical quantum yield of approximately 1.8×10^{-4} can be expected.

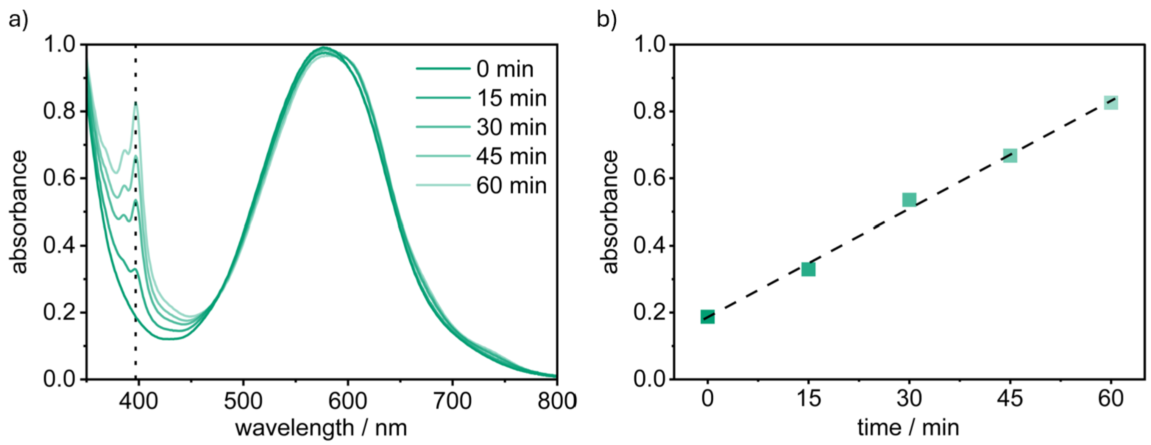

Figure S15: a) Photoredox catalysis experiment identical to that shown in Figure 4D of the main manuscript, performed under standard conditions (100 mM TEOA, 11 mM SDS, 10 mM MV^{2+}, and 80 μM$[Fe(bpyNpy)_2]^{2+}$). Irradiation was carried out using a 150 W xenon arc lamp, with the output passed through a monochromator to isolate 560 ± 10 nm light and a long-pass filter to remove wavelengths below 480 nm. b) Growth of the absorption signal at 395 nm as a function of irradiation time, showing a linear correlation, which indicates that the observed reactivity does not originate from a formed decomposition product.

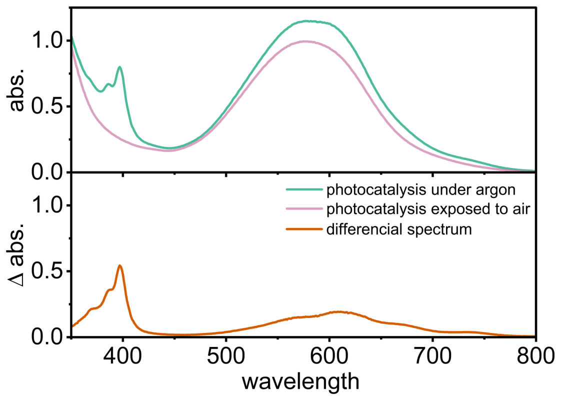

Figure S16: Photoredox catalysis experiment analogous to that shown in Figure 4D of the main manuscript, performed under standard conditions (100 mM TEOA, 11 mM SDS, 20 mM MV^{2+}, and ~100 µM $[Fe(bpyNpy)_2]^{2+}$). After 60 min of irradiation using a 150 W xenon arc lamp, the output of which was passed through a monochromator to isolate 560 ± 10 nm light and a long-pass filter to remove wavelengths below 480 nm, absorption spectra were recorded before (green trace) and after exposure to air (pink trace). Upon exposure to air, the $MV^{•+}$ photoproduct is re-oxidized to MV^{2+}, consistent with oxygen-mediated back-oxidation. Because MV^{2+} does not exhibit absorption bands in the visible region, the spectrum recorded after air exposure is consistent with effective photocatalyst decomposition under prolonged irradiation. The differential spectrum obtained by subtracting the spectrum recorded after air exposure from that recorded under inert conditions (red trace) matches well with the normalized reference spectrum of $MV^{•+}$, confirming that $MV^{•+}$ is formed during photoredox catalysis even though the absorption features between 550 and 650 nm are not immediately apparent in the spectra after 60 min of irradiation (green line).

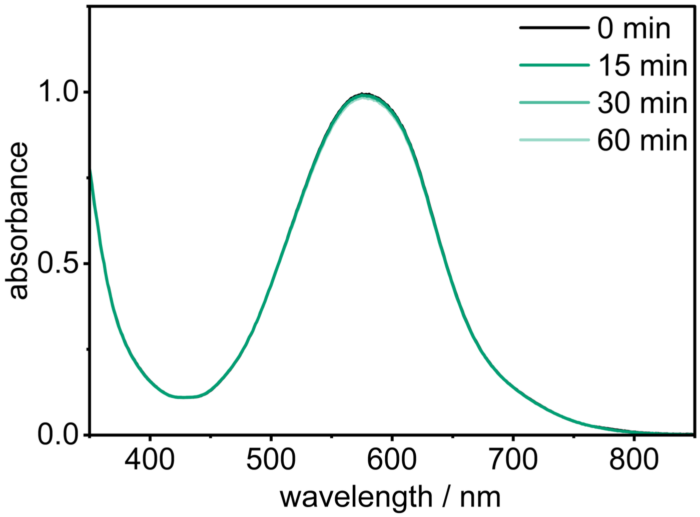

Figure S17: Photostability of $[Fe(bpyNpy)_2]^{2+}$ in water containing 11 mM SDS under irradiation using the same optical setup and conditions as employed for the photocatalysis experiments.

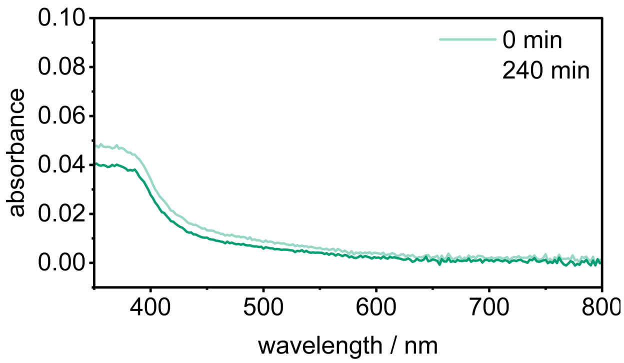

Figure S18: Extended control experiments probing the possible formation of $MV^{\bullet +}$ under prolonged irradiation, since mixtures of MV^{2+} and TEOA can form donor-acceptor pairs that enable photoinduced electron transfer over longer irradiation times. Standard conditions employed 100 mM TEOA, 11 mM SDS, and 10 mM MV^{2+} using a 150 W xenon arc lamp with the output passed through a monochromator to isolate 560 ± 10 nm light and a long-pass filter to remove wavelengths below 480 nm. Under these conditions, no photoinduced electron transfer was observed within 4 hours of irradiation.

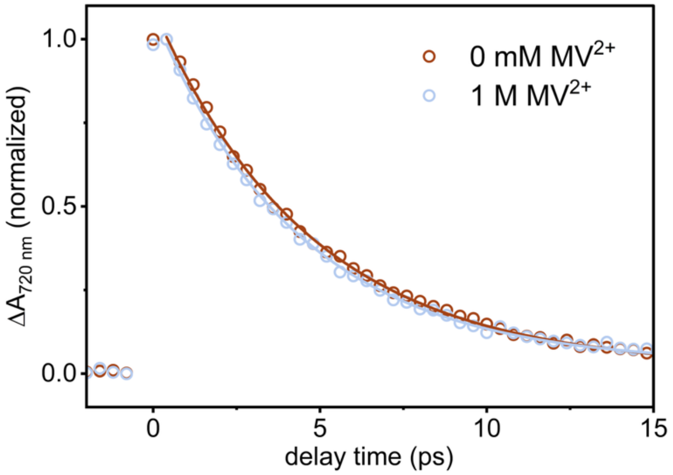

Figure S19: Ultrafast transient absorption kinetics of $[Fe(bpyNpy)_2]^{2+}$ in the absence (brown) and presence (green) of 1 M MV^{2+} in aqueous 11 mM SDS solution. No experimentally significant change in the MLCT excited-state decay kinetics is observed, indicating the absence of productive dynamic quenching.

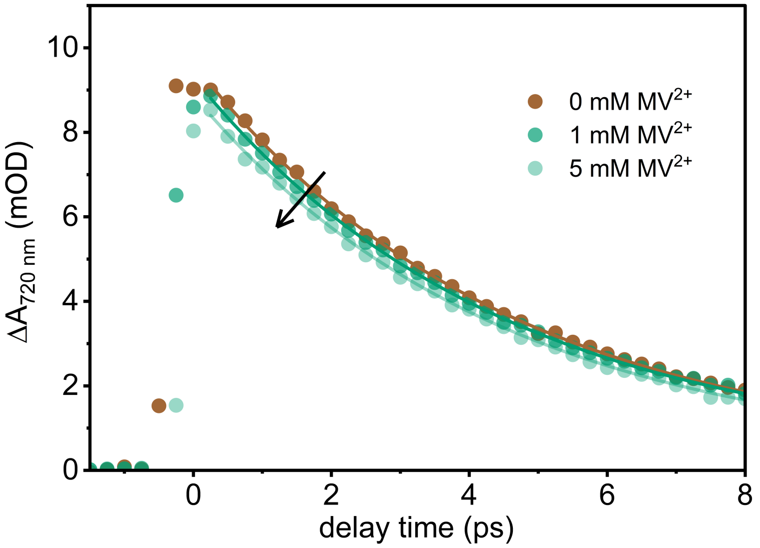

Figure S20: Ultrafast transient absorption kinetics of $[Fe(bpyNpy)_2]^{2+}$ complex in the absence (brown) and presence (green) of 1 mM or 5 mM MV^{2+} quencher in micellar solution. The decrease in MLCT signal amplitude upon addition of MV^{2+} indicates static quenching of the charge-transfer excited state, supporting electron transfer from the ^{3}MLCT state prior to relaxation to the lower-energy 3MC state of the chromophore.

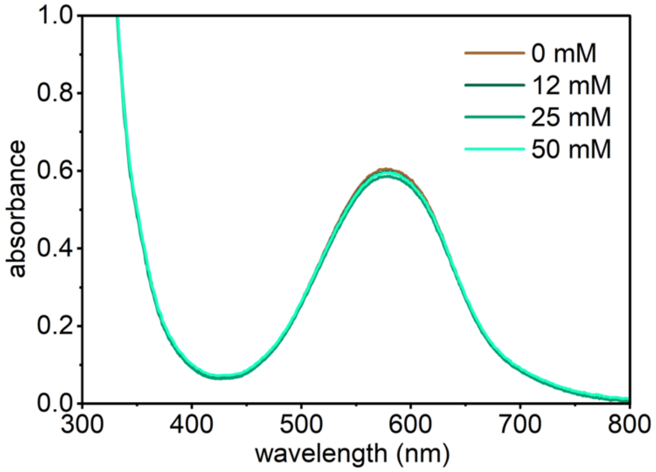

Figure S21: UV-Vis absorption spectra of the sample solutions used for the ultrafast transient absorption measurements shown in Figure S20. No significant changes in the absorption profile are observed upon addition of MV^{2+}, indicating that the variations in transient absorption signal amplitude do not originate from absorption changes upon the addition of MV^{2+} and Cl^{-}.

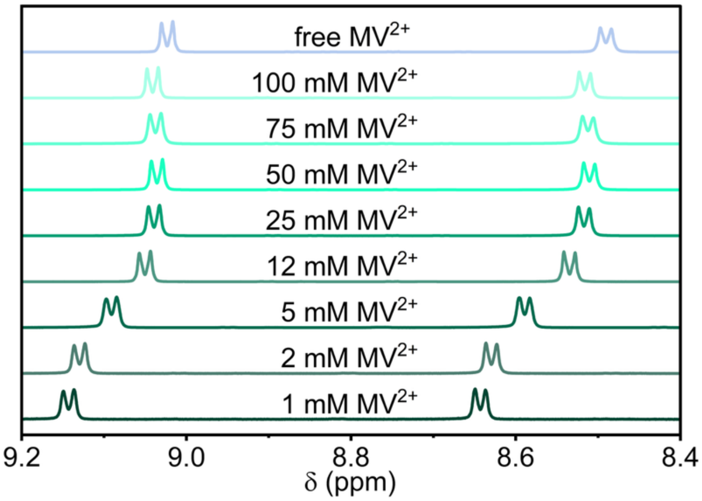

Figure S22: NMR titration experiment of an 11 mM SDS solution in D_2O (blue) upon incremental addition of MV^{2+} (green). Pronounced chemical shift changes at low MV^{2+} concentrations (1-12 mM) indicate progressive incorporation of MV^{2+} into the SDS micellar environment via pre-association. At higher MV^{2+} concentrations (12-100 mM), only minor additional spectral changes are observed, indicating that the pre-association equilibrium is effectively fully shifted toward the associated state.

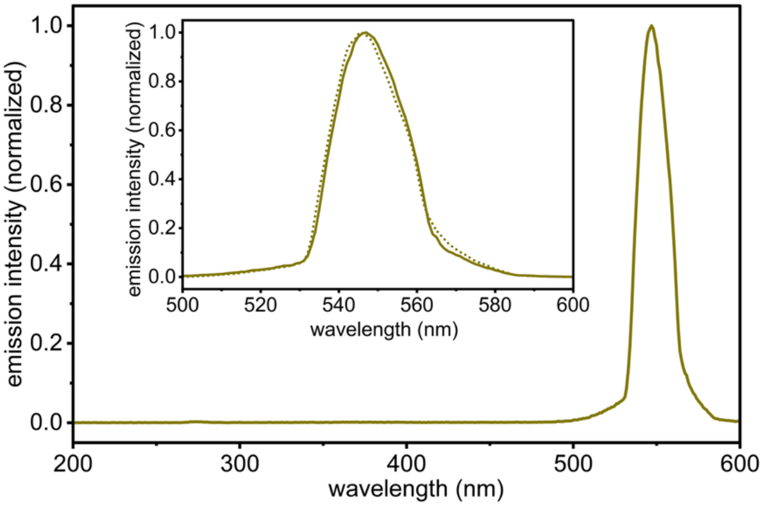

Figure 23: Emission spectrum of the light source used for photoredox catalysis. The solid line shows the emission without the 480 nm long-pass filter, whereas the dotted line shows the emission with the filter applied. The long-pass filter removes shorter wavelengths to prevent undesired high-energy light–driven reactivity, as reported in recent studies.[19,20]

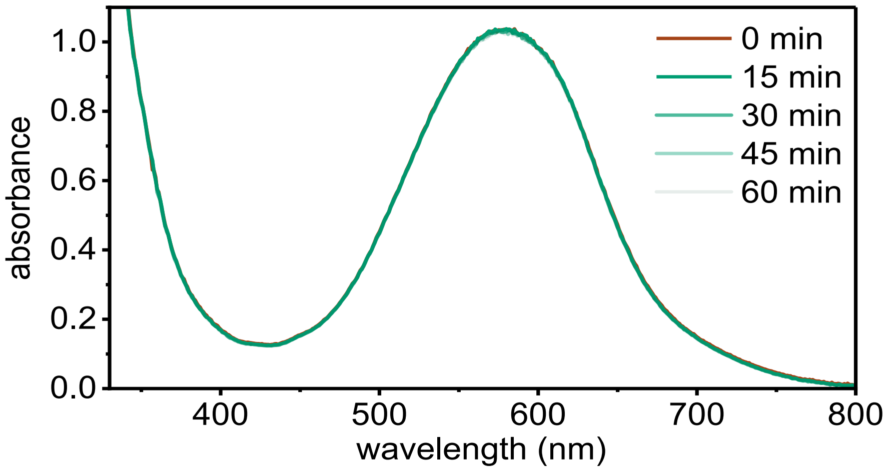

Figure S24: Extended photostability of ~80 μM $[Fe(bpy\textit{N}py)_2]^{2+}$ in deaerated aqueous solution containing 11 mM SDS and 100 mM TEOA under irradiation using the same optical setup and conditions employed for the photocatalysis experiments. No significant spectral changes are observed, indicating that the complex remains stable under the applied reaction conditions.

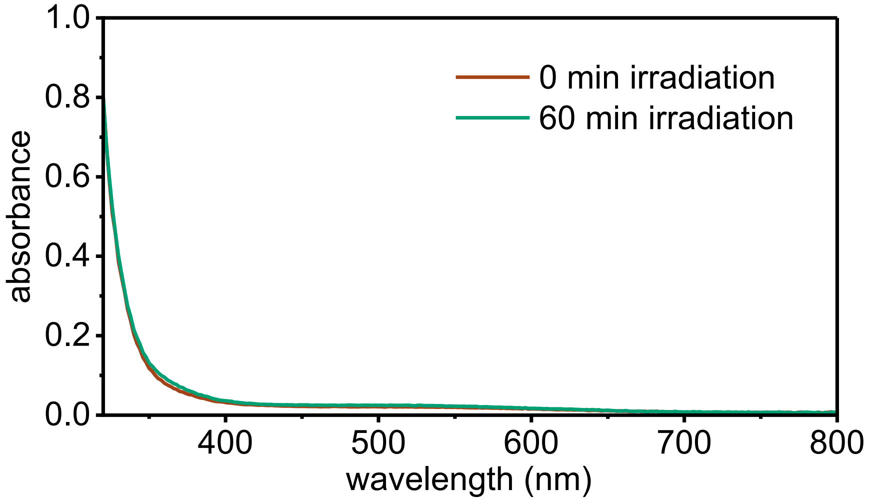

Figure S25: Extended control experiments probing the possible formation of $MV^{\bullet+}$ by oxidation of Fe(II) to Fe(III) upon ligand dissociation. UV-vis absorption spectra of deaerated aqueous sample solutions containing 80 µM $Fe(BF_4)_2$, 180 µM [bpy*N*PyH]PF_6, 10 mM MV^{2+}, 100 mM TEOA, and 11 mM SDS before and after 60 min irradiation with 560 ± 10 nm light from a 150 W xenon arc lamp using a monochromator and a 480 nm long-pass filter. No significant spectral changes are observed, indicating that $MV^{\bullet+}$ does not form through ligand dissociation followed by oxidation of Fe(II) under the applied conditions.

7. References

(1) Fulmer, G. R.; Miller, A. J. M.; Sherden, N. H.; Gottlieb, H. E.; Nudelman, A.; Stoltz, B. M.; Bercaw, J. E.; Goldberg, K. I. NMR Chemical Shifts of Trace Impurities: Common Laboratory Solvents, Organics, and Gases in Deuterated Solvents Relevant to the Organometallic Chemist. *Organometallics* **2010**, *29* (9), 2176–2179.
(2) Carey, M. C.; Adelman, S. L.; McCusker, J. K. Insights into the excited state dynamics of Fe(ii) polypyridyl complexes from variable-temperature ultrafast spectroscopy. *Chem. Sci.* **2019**, *10*, 134–144.
(3) Jamula, L. L.; Brown, A. M.; Guo, D.; McCusker, J. K. Synthesis and characterization of a high-symmetry ferrous polypyridyl complex: approaching the $^5T_2/^3T_1$ crossing point for Fe^{II}. *Inorg. Chem.* **2014**, *53* (1), 15–17.
(4) Snellenburg, J. J.; Laptenok, S.; Seger, R.; Mullen, K. M.; van Stokkum, I. H. M. Glotaran: A Java-Based Graphical User Interface for the R Package TIMP. *J. Stat. Softw.* **2012**, *49*, 1–22.
(5) Brown, A. M.; McCusker, C. E.; Carey, M. C.; Blanco-Rodriguez, A. M.; Towrie, M.; Clark, I. P.; Vlcek, A.; McCusker, J. K. Vibrational Relaxation and Redistribution Dynamics in Ruthenium(II) Polypyridyl-Based Charge-Transfer Excited States: A Combined Ultrafast Electronic and Infrared Absorption Study. *J. Phys. Chem. A.* **2018**, *122* (40), 7941–7953.
(6) Bugaenko, D. I.; Yurovskaya, M. A.; Karchava, A. V. Reaction of Pyridine-N-Oxides with Tertiary sp^2-*N*-Nucleophiles: An Efficient Synthesis of Precursors for *N*-(Pyrid-2-yl)-Substituted *N*-Heterocyclic Carbenes. *Adv. Synth. Catal.* **2020**, *362* (24), 5777–5782.
(7) Koizumi, T.; Tomon, T.; Tanaka, K. Synthesis and electrochemical properties of bis(bipyridine)ruthenium(II) complexes bearing pyridinyl- and pyridinylidene ligands induced by cyclometalation of N′-methylated bipyridinium analogs. *J. Organomet. Chem.* **2005**, *690*, 1258–1264.
(8) Brown, A. M.; McCusker, C. E.; McCusker, J. K. Spectroelectrochemical identification of charge-transfer excited states in transition metal-based polypyridyl complexes. *Dalton Trans.* **2014**, *43* (47), 17635–17646.
(9) Reinhard, M. E.; Sidhu, B. K.; Lozada, I. B.; Powers-Riggs, N.; Ortiz, R. J.; Lim, H.; Nickel, R.; Lierop, J. V.; Alonso-Mori, R.; Chollet, M.; et al. Time-Resolved X-ray Emission Spectroscopy and Synthetic High-Spin Model Complexes Resolve Ambiguities in Excited-State Assignments of Transition-Metal Chromophores: A Case Study of Fe-Amido Complexes. *J. Am. Chem. Soc.* **2024**, *146*, 17908–17916.
(10) Ghosh, A.; Yarranton, J. T.; McCusker, J. K. Establishing the origin of Marcus-inverted-region behaviour in the excited-state dynamics of cobalt(III) polypyridyl complexes. *Nat. Chem.* **2024**, *16*, 1665–1672.
(11) McCusker, J. K.; Rheingold, A. L.; Hendrickson, D. N. Variable-Temperature Studies of Laser-Initiated $^5T_2 \rightarrow {}^1A_1$ Intersystem Crossing in Spin-Crossover Complexes: Empirical Correlations between Activation Parameters and Ligand Structure in a Series of Polypyridyl Ferrous Complexes. *Inorg. Chem.* **1996**, *35*, 2100–2112.
(12) Perinelli, D. R.; Cespi, M.; Lorusso, N.; Palmieri, G. F.; Bonacucina, G.; Blasi, P. Surfactant Self-Assembling and Critical Micelle Concentration: One Approach Fits All? *Langmuir* **2020**, *36* (21), 5745–5753.
(13) Ingram, T.; Storm, S.; Kloss, L.; Mehling, T.; Jakobtorweihen, S.; Smirnova, I. Prediction of micelle/water and liposome/water partition coefficients based on molecular dynamics simulations, COSMO-RS, and COSMOmic. *Langmuir* **2013**, *29* (11), 3527–3537.

(14) Anand, U.; Jash, C.; Mukherjee, S. Spectroscopic determination of Critical Micelle Concentration in aqueous and non-aqueous media using a non-invasive method. *J. Colloid. Interface Sci.* **2011**, *364* (2), 400–406.
(15) Ishkhanyan, H.; Rhys, N. H.; Barlow, D. J.; Lawrence, M. J.; Lorenz, C. D. Impact of drug aggregation on the structural and dynamic properties of Triton X-100 micelles. *Nanoscale* **2022**, *14* (14), 5392–5403.
(16) Varade, D.; Joshi, T.; Aswal, V. K.; Goyal, P. S.; Hassan, P. A.; Bahadur, P. Effect of salt on the micelles of cetyl pyridinium chloride. *Coll. Surf. A, Phys. Chem. Eng. Asp.* **2005**, *259* (1-3), 95–101.
(17) Radjagobalou, R.; Blanco, J.-F.; Dias da Silva Freitas, V.; Supplis, C.; Gros, F.; Dechy-Cabaret, O.; Loubière, K. A revised experimental protocol for implementing the actinometry method with the Reinecke's salt. *J. Photochem. Photobiol. A* **2019**, *382*.
(18) Montalti, M.; Credi, A.; Prodi, L.; Gandolfi, T. M. *Handbook of Photochemistry*; CRC/Taylor & Francis: Boca Raton, 2006.
(19) J. A.Moghtader, M.-S.Bertrams, D.Schollmeyer, C.Kerzig, Angew. Chem. Int. Ed.**2025**, 64, e202509203.
(20) N.Beaucage, Z.Singh, J.Bourdon, S. K.Collins, Angew. Chem. Int. Ed.**2025**, 64, e202412606.
